# Artificial Intelligence and Metazoan Nervous Systems: From Local Processing to Valenced States of the Organism and the Inference of Suffering

**DOI:** 10.3390/ani16213376

**Published:** 2026-10-26

**Authors:** Çağrı Çağlar Sinmez, René van den Hoven, Tünay Karan, Ali Cesur Onmaz, Kübra İpek Özek

**Affiliations:** 1 Department of History of Veterinary Medicine and Deontology, Faculty of Veterinary Medicine, Erciyes University, Kayseri 38280, Türkiye; cagrisinmez@erciyes.edu.tr; 2 Clinical Unit of Equine Internal Medicine, Clinical Centre for Equine Health and Research, University of Veterinary Medicine Vienna, 1210 Vienna, Austria; prof.renevandenhoven@gmail.com; 3 Department of Genetics, Faculty of Veterinary Medicine, Yozgat Bozok University, Yozgat 66100, Türkiye; 4 Department of Internal Medicine, Faculty of Veterinary Medicine, Erciyes University, Kayseri 38280, Türkiye; aconmaz@erciyes.edu.tr; 5 Department of Mechanical Engineering, Faculty of Engineering-Architecture, Yozgat Bozok University, Yozgat 66100, Türkiye; kubraipek.ozek@yobu.edu.tr

**Keywords:** affective neuroscience, animal welfare, artificial intelligence, biological autonomy, comparative neuroscience, domestic animals, neuromodulation, sentience, suffering, volume transmission, good health and well-being

## Abstract

Recognising animals as sentient does not establish whether an individual animal is suffering at a particular time. This structured critical narrative review examines how biological evidence can support that inference. Drawing on evolutionary and comparative neuroscience, animal-welfare research and studies of artificial systems, we examine proposed links among stimulus detection, wider neural modulation, valuation, recurrent integration and consequences for the whole organism. Artificial systems provide comparisons in which learning and adaptive processing can be implemented without independently establishing subjective experience. Their implementation neither establishes feeling nor shows that these functions are unnecessary in conscious animals. Amphioxus, zebrafish, birds, mammals and octopus illustrate distinct questions about neural organisation, internal-state regulation and valuation. Birch et al.’s dimensions guide comparison of possible forms of experience without ranking species on a single scale. Exploratory literature searches, a targeted PubMed audit and a supplementary Scopus coverage check informed source identification and verification. Evidence was appraised separately for each claim, considering alternative explanations, marker validity, dependence among measures, interventions and changes over time. The framework distinguishes capacity for experience, occurrence of a particular state and its welfare significance while assessing confidence in each inference. The proposed biological links organise this assessment; they are not a validated causal sequence or scale of consciousness. Both brief severe experiences and sustained adverse states warrant consideration. AI can extend observation, but its classifications require independent biological validation and evaluation under their intended conditions of use.

## Simple Summary

An animal’s immediate reaction to a stimulus does not, by itself, tell us what the animal feels. This review examines how information about the environment and the body influences behaviour, learning and states that may continue after an event has ended. Comparisons with artificial systems help explain why complex performance alone does not establish feeling. Studies of amphioxus, zebrafish, birds, mammals and octopus illustrate what different experiments can reveal while leaving important questions about experience unresolved. We consider possible differences between animal experiences without placing species on a single evolutionary ladder. Assessment must distinguish whether an animal could have an experience, whether it is occurring now and how seriously it affects the animal. Both brief severe experiences and persistent or recurring negative states may impose substantial welfare burdens. Confidence in the interpretation should be reported separately from the level of welfare concern. Artificial intelligence can improve observation, but its outputs still require biological interpretation.

## 1. Introduction: From Legal Sentience to Biological Evidence

European animal-welfare law recognises animals as sentient beings. Article 13 of the Treaty on the Functioning of the European Union requires the Union and its Member States to take animal welfare fully into account while respecting national provisions and customs concerning religious rites, cultural traditions and regional heritage [1]. This creates a legal duty to consider welfare alongside other interests. It does not define sentience biologically, show that every aversive event causes suffering or decide whether a particular use of animals is acceptable.

This review examines how observations of an animal can support an inference that it is experiencing a negative state and when that state may amount to suffering. We distinguish the capacity for experience from evidence that an experience is occurring and from the burden it may impose. We follow the relevant biological processes from stimulus detection to changes in internal state, valuation, integration and consequences for the whole animal. Comparisons with artificial systems help clarify what these functions can-and cannot tell us about feeling.

Sentience is the capacity for positive or negative experience [2]. It does not show that a particular animal is suffering at a particular time [3,4]. That conclusion requires evidence about the animal’s present state. Behaviour, physiology and context must be interpreted together, with attention to the severity and duration of any negative state [5]. A withdrawal reflex, brief startle, exertion or temporary frustration may be biologically important without establishing pain or sustained negative affect [6,7].

Artificial intelligence sharpens this distinction because sophisticated performance does not by itself establish experience. Artificial systems can learn, predict, classify and produce adaptive outputs, including language or behaviour that resembles emotional communication. Biological and artificial neural networks can both learn complex relationships directly from data without first reducing them to simple rules [8]. This functional similarity makes comparison useful, but it does not establish similarity in biological organisation or subjective experience. The relevance to welfare assessment is inferential: an observable function may have several explanations, so its interpretation requires evidence about the animal’s biological state.

In animal-welfare assessment, behaviour, facial expression, avoidance, vocalisation, task performance and physiological change are observable signs, not pain or suffering themselves. Avoidance may arise from nociception, fear, learning or reflex organisation. Facial changes may reflect not only pain but also fatigue, arousal, disease or context. Heart rate, cortisol and immune responses show biological challenge without revealing how that challenge is experienced [6,9,10,11]. Reliable assessment therefore depends on partly independent evidence, species-specific context, and information about the duration and reversibility of the observed changes.

Arshavsky has argued more strongly that animal consciousness cannot be inferred from behaviour alone and that neurophysiological mechanisms are required [12]. Behaviour can contribute to an assessment of consciousness when considered alongside other biological evidence. Similar behaviour alone, however, does not establish similar experience in animals or AI.

Reviews of behavioural pain scales and facial-expression scoring systems for farm animals show that validation has focused mainly on observer agreement, responsiveness and discrimination between presumed painful and control conditions. Of 20 pain-assessment tools assessed in one systematic review, 13 had low or very low overall strength of evidence; a separate review of grimace scales found that several forms of reliability and validity remained understudied [13,14]. These tools can improve clinical detection, but they do not calibrate a score against the magnitude of subjective experience. The wider science of consciousness faces the same gap between an observable proxy and the inaccessible experience it is used to infer [15].

We first examine the evolutionary origins of neural regulation and the mechanisms through which a local event can influence the wider organism. Comparative examples then show what different experiments establish about modulation, valuation, integration and persistence. From these findings, we develop rules for assessing welfare-related evidence, examine their implications for comparisons with artificial systems, and consider their application to domestic animals and automated observation.

Table 1 defines the key terms used in this review. Terms such as affect, arousal, valence, suffering and consciousness do not always have the same meaning in neuroscience, animal-welfare science and artificial-intelligence research. Our working definitions distinguish five things: an observable response, an internal biological state, positive or negative evaluation, conscious feeling, and confidence in a welfare inference. They are intended to make the argument clear, not to serve as universal definitions.

## 2. Materials and Methods

### 2.1. Review Design

We conducted a structured critical narrative review, not a systematic review or meta-analysis. We combined evidence from evolutionary neurobiology, affective and comparative neuroscience, animal-welfare science, veterinary and behavioural research, and studies of artificial networks. For each type of evidence, we asked two questions: what conclusion does the measurement support, and where does interpretation go beyond the measurement? An exploratory search identified relevant mechanisms and recurring problems of inference. We then checked the central claims against their cited sources. We also asked whether each proposed mechanism could be implemented in an artificial system without independent evidence of subjective experience. If so, we treated that mechanism as evidence of information processing or adaptive control but not as sufficient evidence of feeling.

### 2.2. Exploratory Literature Identification and Keyword Development

We identified literature through repeated searches of PubMed/MEDLINE (National Library of Medicine, Bethesda, MD, USA), Scopus (Elsevier, Amsterdam, The Netherlands), Google Scholar (Google LLC, Mountain View, CA, USA) and publisher databases. We also followed references backward and forward from key papers. Search terms covered the biological sequence examined in this review: the evolution of cellular signalling; volume transmission and neuromodulation; arousal, affect, pain, sentience and consciousness; recurrent processing, memory, flexible integration and network organisation; autonomic, endocrine, metabolic and immune links; recovery; and artificial neural networks as comparison cases. We added synonyms and indexing terms found in titles and abstracts. Known relevant papers informed the refinement of search terms. Because terminology differs across these fields, these searches were used to identify and verify sources, not to estimate how often a topic had been published.

On 16 October 2026, we supplemented source discovery with five focused Scopus searches addressing comparative emotion and inference, neuromodulation and internal states, valuation and relief, artificial-system consciousness indicators, and validation of automated animal assessment. The search author reported that no filters were applied on the results pages before export and that all results were exported. The five exports contained 15,512 record appearances, matching the reported Scopus totals. Deduplication by Scopus identifier and matching DOI retained 15,360 records.

Automated prioritisation and comparison with the existing bibliography guided targeted appraisal of potentially consequential omissions. Selected sources were assessed for whether they strengthened, qualified or challenged specific manuscript claims. This was an exploratory coverage check, not an exhaustive systematic screening of all retrieved records. Supplementary Material S1 documents the supplied search history, export details, deduplication procedure and appraisal approach.

### 2.3. Targeted PubMed Coverage Audit

On 22 July 2026, we performed a focused PubMed audit to see how often four predefined topics occurred together in titles, abstracts and indexing terms. The four search blocks covered: (A) domestic or farm animals; (B) welfare, affect, pain, suffering, sentience or consciousness; (C) neurobiology or neurophysiology; and (D) frameworks, inference, validation, evidence integration or decision support. We applied no filters for date, language, article type or full text. Searches combining A + B, A + B + C, A + B + D and A + B + C + D returned 31,514, 3607, 249 and 25 records, respectively. Without the animal block, B + C + D returned 2416 records. Supplementary Material S1 contains the complete search strings, PubMed history and search date. This audit cannot establish that related frameworks are absent, estimate their prevalence or prove the novelty of our approach.

One author screened the titles and abstracts of all 25 records found by the four-block search. There was no duplicate screening or formal test of agreement between reviewers. A record met all four criteria only if it addressed domestic animals, welfare-related affect or suffering, neurobiological evidence, and an explicit method for turning that evidence into an inference or decision. Twenty records were clinical, psychometric or intervention studies. Three made a limited link between an observable measure and affect or consciousness, one used the Five Domains Model, and one was retrieved because of terminology rather than content. None met all four criteria. We report this only as a finding within a narrow PubMed search. Related concepts and frameworks exist outside this intersection and were included through the broader searches and citation tracking.

### 2.4. Claim-Level Appraisal and Synthesis

We assessed evidence separately for each claim rather than giving each paper one overall quality score. Construct validation asks whether an indicator reflects the state it is intended to assess. Mason [16] describes complementary validation tests that rely on different assumptions. Confidence in an indicator increases when it passes tests with different potential sources of error.

We first separated what the study measured from what the authors inferred. We then considered whether the measure was biologically specific, validated in the relevant species and setting, open to alternative explanations, supported by an intervention or follow-up over time, and independent of other measures used in the same inference. We re-examined evidence cited for theories instead of accepting it because proponents had cited it [17]. We also examined artificial implementations of the same function as comparison cases, without presuming that the systems were non-conscious. Such comparisons help identify alternative functional explanations; they do not, by themselves, establish or exclude subjective experience. This does not mean that the function is unnecessary for consciousness in a suitable biological system. Each finding was used to address one or more of four questions: Can the species experience such a state? Is the state occurring now? What burden does it impose on the animal? How confident can we be in these conclusions? The evidence came from diverse disciplines and study designs, so we used a narrative synthesis and did not calculate a combined score for consciousness or emotional intensity.

Evidence was appraised separately for each inference: stimulus detection, distributed state modulation, biological valuation, flexible integration, and welfare-relevant burden. A finding could inform more than one inference, but support for one did not automatically establish the others. For each claim, we distinguished the measured result, its proposed interpretation, competing explanations, and the remaining uncertainty. Convergent findings were given greater weight when they constrained different alternatives, rather than providing correlated measures of the same response. Missing evidence, an uninformative negative result, and evidence contradicting a claim were distinguished. The proposed links between these processes constitute a biological synthesis; they have not been validated as a complete causal sequence or as a scale of subjective experience.

### 2.5. Use of AI-Assisted Tools

Generative AI tools (ChatGPT (GPT-5, OpenAI, San Francisco, CA, USA)/Claude (Anthropic, San Francisco, CA, USA) supported keyword development, record parsing, deduplication, topic prioritisation, bibliography comparison and preparation of draft appraisal rationales. They also helped organise claim-evidence tables and compare selected source passages with manuscript claims. An author ran the PubMed searches. The authors reviewed the AI-assisted recommendations and made the final decisions on source interpretation, inclusion and manuscript wording. Supplementary Material S1 describes the search and appraisal procedures and the role of AI assistance.

### 2.6. Species Selection and Comparative Rationale

Species were selected purposively to examine distinct components of the proposed framework rather than to represent animal diversity or establish a ranking of consciousness. Amphioxus was considered in relation to central neural coordination and the evidence required to infer positive or negative valuation. Zebrafish studies were used to examine how long changes in neural activity or behaviour last, how drugs alter these responses, and how earlier events influence later behaviour. Birds and mammals were used to examine recurrent integration associated with affect across different forebrain organisations, including systems with and without a laminated mammalian neocortex. Octopus was included as a comparative case involving an independently evolved nervous system with substantial distributed processing in the arms. These cases were selected for their relevance to the review’s specific inferential questions, not to rank species by brain size, similarity to humans or presumed level of consciousness.

## 3. Evolutionary Origins of Integrated Biological Regulation

Intercellular communication predates nervous systems. Nervous systems evolved from older signalling mechanisms and enabled faster, more selective coordination across the body [18,19]. Multicellular animals arose more than 600 million years ago, and early neural systems probably appeared in the late Ediacaran, before the Cambrian began about 539 million years ago. Even before centralised brains existed, animals had to coordinate movement, feeding, defence and internal regulation across many cells.

Several capacities later incorporated into nervous systems were already present in non-neural cells, including excitability, secretion, membrane gradients, persistent responses and discrimination between stimuli [19,20,21]. Voltage-gated ion channels predate animals and were later recruited for neural signalling, muscle contraction and secretion [22]. Presynaptic release machinery developed from older calcium-regulated vesicle systems, while postsynaptic complexes incorporated sensory and adhesion components [23]. A biological neuron is therefore not a primitive computational point but a specialised living cell assembled from older regulatory modules.

Diffuse chemical signalling also predates nervous systems and later contributed to neuronal volume transmission. At least 30 neuropeptide systems can be traced to the common ancestor of protostomes and deuterostomes [24]. *Hydra* shows how an early nerve net could combine peptide signalling with relatively rapid transmission through peptide-gated ion channels [25]. As metazoan lineages diversified during the late Ediacaran and early Cambrian, they produced many combinations of cellular coordination, nerve nets, ganglia, sensory specialisation and centralisation. Evolution did not follow a single ladder from simple animals to humans.

Placozoans lack neurons and synapses, yet their simple form conceals substantial cellular, chemical and evolutionary complexity [26]. Cnidarian nerve nets can likewise contain diverse peptide systems [25]. Chordates appeared more than 500 million years ago. Amphioxus retains homologues of several vertebrate neural features, whereas the telencephalon and neural crest were important innovations near the origin of vertebrates [27].

**Figure 1:**
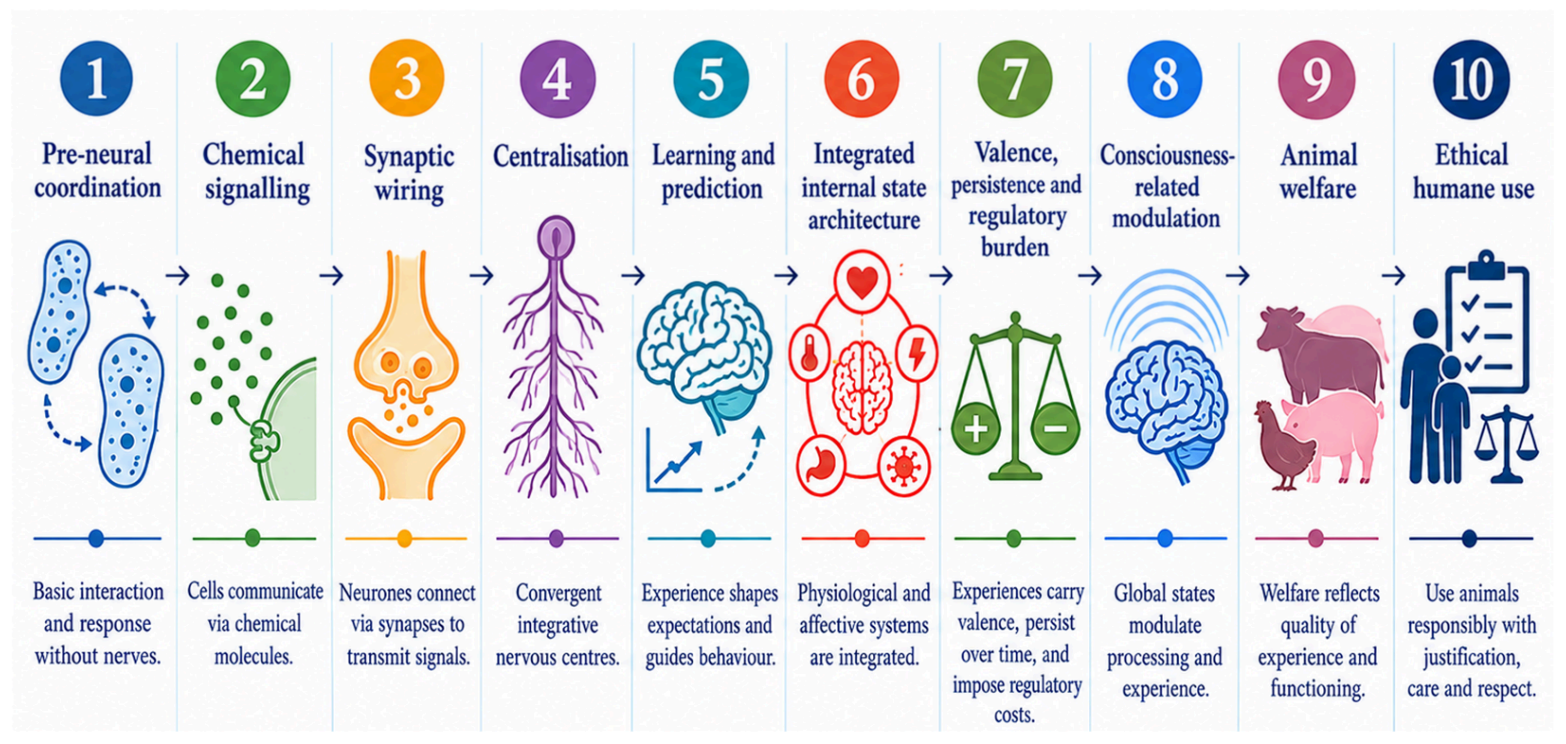
From primitive coordination to animal welfare. The figure presents a conceptual progression from non-neural coordination and chemical signalling to synaptic wiring, centralisation, learning, integrated internal-state regulation, valence, possible conscious modulation, animal welfare and ethical human use. It is a heuristic sequence, not a strict phylogeny or a ranking of species. The sequence organises questions about coordination, valuation, possible experience and welfare; it does not establish an evolutionary threshold for suffering.

Cognition and affective regulation did not appear in one step. Older mechanisms were repeatedly modified and linked into broader systems that regulated the whole organism [28] (Figure 1).

This evolutionary history matters when nervous systems are compared with AI [29,30]. Artificial networks are built to convert inputs into outputs. Nervous systems evolved inside organisms that already had to maintain metabolism, bodily boundaries, repair and reproduction. Shared molecules can demonstrate common ancestry but do not by themselves demonstrate similar cognition or experience [31]. The important question is how these mechanisms work together in the living animal.

## 4. From Point-to-Point Signalling to Distributed State Modulation

Neural communication is not limited to point-to-point synapses. Agnati and colleagues distinguished wiring transmission from volume transmission [32]. Wiring transmission follows defined routes through axons, chemical or electrical synapses and close membrane contacts. In volume transmission, chemical or physical signals spread through extracellular fluid or cerebrospinal fluid and influence more distant targets.

The two modes differ in speed, precision and reach. Wiring transmission can deliver rapid, spatially precise signals. Volume transmission can influence many cells over a wider area and for longer. In the serotonergic system, transmitter released from non-junctional varicosities reaches extrasynaptic receptors and glia, allowing relatively few neurons to influence plasticity, circadian rhythms, feeding, social behaviour and mood-related states [33]. Neuropeptides released with classical transmitters become especially important during pain, stress, injury and prolonged challenge [34]. Electrical and mixed chemical–electrical synapses provide further ways to synchronise neural populations [35].

Diffuse neuromodulation can therefore turn a local event into a change in the conditions under which later information is processed. It can alter salience, attention, plasticity, motivation and response priorities. Dopaminergic control of basal-ganglia-like circuits was already present in lampreys, whose lineage diverged from jawed vertebrates more than 500 million years ago [36]. State-dependent modulation is thus evolutionarily ancient.

Wiring and volume transmission are not successive stages, and a nervous system does not have to use only one of them. Peptide signals existed before neurons and synapses. As nervous systems became more centralised, these signals were incorporated into more localised and circuit-specific systems. A single neuron can release classical neurotransmitters and neuropeptides in different proportions, depending on how strongly and for how long it fires. This chemical multiplexing allows the same anatomical network to operate differently over seconds, minutes or longer periods and across small or large regions [37,38]. It is therefore a plausible mechanism for persistent, state-dependent regulation. On its own, however, it does not demonstrate positive or negative feeling or conscious experience.

Diffuse signalling is not a marker of consciousness. Artificial networks can use diffusion-like signals to create task-specific modules, reduce the loss of previously learned information and improve learning when feedback is delayed or sparse [39,40]. Earlier models also showed that diffusible signals can organise learning in both biological and artificial networks [41]. These examples show why diffusion alone does not establish subjective experience, even though diffuse neuromodulation may contribute to that experience in animals.

The number of terminals or receptors involved in volume transmission is therefore not a measure of consciousness. The more useful questions are: How far does the signal spread? How long does it act? Which cells and receptors respond? Does it recruit affective or autonomic systems? Does it interact with thalamic or pallial networks? Does it change later behaviour or physiological regulation? Volume transmission is best viewed as one mechanism by which a local signal can alter a wider neural state, not as the location of feeling. The next section examines the evidence needed to distinguish distributed modulation from biological valuation, flexible integration and adverse consequences for the animal.

## 5. From a Local Neural Event to a Valenced Organismal State

### 5.1. Local Detection and Rapid Response

A noxious stimulus can elicit a withdrawal reflex without that response, by itself, establishing conscious pain [42]. Human perceptual studies also show that early sensory processing can occur without conscious access [7,43,44].

Spinal and brainstem circuits can generate and adjust complex motor patterns without requiring moment-to-moment conscious specification of each movement. In locomotion, central pattern-generating circuits organise rhythmic movement and integrate sensory feedback, while descending pathways initiate and modify the pattern [45,46]. Coordinated movement therefore demonstrates motor control but does not by itself establish pain or suffering.

Arousal, motor activity, consciousness and affective valence must also be distinguished. Ascending pathways from the brainstem help maintain wakefulness. Wakefulness alone, however, does not tell us whether or what an animal consciously experiences [43]. Equine pharmacology makes the distinction concrete. Opioids and dopaminergic stimulants can increase locomotion or repetitive activity, whereas sedatives can markedly reduce movement without necessarily abolishing perception [47,48,49]. Tobin’s contrast between horses “running happy” and “running scared” illustrates the problem: similar locomotion may result from analgesia, disinhibition, motor stimulation, hyper-responsiveness or fear [47]. Running is directly observed; its affective meaning is inferred.

Behavioural activation cannot therefore be read as welfare-positive or welfare-negative by itself. Increased movement may reflect exploration, play, panic, pain, escape or pharmacological stimulation; reduced movement may reflect calmness, fatigue, sedation, illness or freezing. Arousal indicates activation, but its welfare meaning depends on valence, context, controllability, persistence and recovery. Human aesthetic chills illustrate this distinction: shivers and goosebumps can accompany rewarding or threatening stimuli. Their relationship to interoception, prediction and valuation remains an active area of investigation [50].

### 5.2. Distributed Modulation of Neural State

A sensory event can activate neuromodulatory systems that change how the animal responds to later stimuli, including those detected through another sense. Whether these changes persist after the initiating event requires evidence from measurements over time and targeted interventions.

Wiring and volume transmission are coexisting modes of neural communication [32,33], while neuropeptides can modify signalling alongside classical neurotransmitters [34]. Whether these mechanisms sustain a state after the initiating event requires temporal and intervention evidence.

In larval zebrafish, a 30 s exposure to water flow increased locomotor activity for several minutes and enhanced responsiveness to weak visual-motion stimuli. Calcium imaging revealed increased activity in serotonergic dorsal raphe neurons after flow exposure. Genetic ablation of these neurons abolished the enhancement of visual responsiveness without affecting baseline visual function or locomotor activity [51]. These findings support a causal contribution of dorsal raphe neurons to state-dependent sensory modulation: an event in one sensory modality changed subsequent responsiveness in another. They do not, by themselves, establish negative valence or conscious experience.

Artificial diffusion models provide a complementary functional comparison. Diffusion-based modulation can support task-specific learning [39] and, in a preprint model, temporal credit assignment [40]. These results concern learning mechanisms, not evidence of subjective experience, and do not negate the biological contribution of neuromodulation. The next question is whether the altered state changes the value of events and available actions for the animal.

### 5.3. Affective Valuation and Motivation

In this framework, affect refers to positively or negatively valenced states that influence motivation, learning and action, without assuming that they are consciously felt. Emotional states can therefore be studied through behavioural, physiological, neural and cognitive changes [52]. Approach, avoidance, reinforcement and later choices help reveal what an animal values positively or negatively. Converging behavioural and neural evidence may strengthen an inference of feeling, but the conclusion still depends on how consciousness is understood [4,52].

Cabanac’s proposal that pleasure provides a common currency for weighing competing needs and behavioural options illustrates the step from valuation to feeling [53]. It offers an explanation of value-based choice without demonstrating that every such choice involves conscious pleasure. Other frameworks focus more directly on how affective states function. Mendl and colleagues integrate valence, arousal, short-term emotional responses, longer-term mood and decision-making [54]. Anderson and Adolphs propose studying internal emotion states through shared functional properties and their causal relationships to behaviour across species [55]. These approaches provide precedents for examining valuation separately from its possible subjective component.

Motivational trade-offs offer another way to investigate valuation by testing whether an animal accepts a risk of harm to obtain a reward. Brown and Birch [56] argue that the significance of such choices for sentience depends on the nature of the trade-off and its underlying neural mechanisms. Such choices provide relevant evidence but do not establish feeling on their own.

Stimulation studies bring this distinction into experimental focus. Panksepp and colleagues discuss how electrical stimulation of subcortical brain regions elicits organised behavioural responses and produces rewarding or aversive effects. Their review presents the affective neuroscience interpretation of these findings as evidence of basic emotional feelings alongside alternative cognitive neuroscience accounts [57]. In our framework, these findings support affective valuation, while the attribution of feeling requires further justification.

The relevant mechanisms also extend beyond individual brain regions. The central autonomic network links bodily regulation with cognitive and emotional processing [58], while interacting circuits involving the amygdala and periaqueductal grey contribute to defensive behaviour [59]. These findings support a network account of emotional and cognitive processing [60], without identifying a sufficient neural basis for conscious feeling.

Conditioned place preference or aversion provides a complementary behavioural approach by testing how an earlier event influences later choices. Ede et al. compared calves’ preferences for pens associated with disbudding under local anaesthesia, with or without additional post-procedural analgesia. Calves spent more time and lay down more frequently in the pen associated with local anaesthesia plus meloxicam than in the pen associated with local anaesthesia alone. In contrast, calves spent less time in the pen associated with local anaesthesia plus ketoprofen than in the pen associated with local anaesthesia alone. The authors suggested that ketoprofen may have worn off during conditioning, allowing pain to increase towards its end, but did not directly test this explanation [61].

These learned, treatment-dependent preferences support differences in the negative value associated with the post-procedural conditions. They provide indirect evidence of the affective component of pain, while conscious feeling remains an inference. Place conditioning can also yield negative results. Ede et al. [62] found no subsequent avoidance of the pen associated with castration performed under multimodal pain control. Pain control and features of the conditioning procedure could explain this finding; the absence of avoidance does not establish that pain was absent.

### 5.4. Recurrent Integration, Flexible Use and the Limits of Network Markers

Conscious access is often linked to recurrent activity: neural signals circulate through connected regions instead of passing once through a feed-forward pathway. This can keep selected information available for attention, memory, evaluation and flexible action. The global neuronal workspace theory describes the wider distribution of this information as global broadcasting. In human experiments with brief sensory stimuli, neural activity associated with conscious access often appears relatively late, sometimes about 300 ms after stimulus onset [43,44]. This is not a universal cut-off; timing depends on the sense tested, the task and the experimental design.

Here, access refers to the availability of information for reasoning and the control of action or report; phenomenal consciousness concerns subjective experience. These concepts must be distinguished when interpreting functional evidence [63]. Some forms of information integration can occur without awareness, so the kind and conditions of integration matter more than its mere presence [64].

Within this framework, information that persists, interacts with memory and can guide more than one possible action provides stronger grounds for investigating conscious availability than a brief, stereotyped reflex.

Comparative assessment should not assume that conscious processing requires the specific organisation of the human neocortex [65]. Birch and colleagues propose assessing animal consciousness across multiple dimensions [66]. Different anatomical arrangements may support comparable functions, but functional similarity alone does not establish equivalent subjective experience.

No current theory provides an agreed test of consciousness across species. Theories propose different mechanisms and explain different aspects of consciousness [67]. Evidence presented in their support does not always remain convincing when independently re-examined [17]. We therefore do not rely on one theory or treat recurrence, broadcasting or network complexity as a decisive marker. Instead, we ask what each finding can establish about an animal’s capacity for experience, its current state and the burden that state may impose. An inference of suffering becomes stronger when complementary evidence supports a negatively felt experience and limits plausible alternative explanations. In this assessment, recurrent processing is considered alongside memory, affective evaluation and flexible action as potentially relevant to conscious experience. These features guide assessment rather than constitute a validated cross-species test of consciousness [52,65].

**Figure 2:**
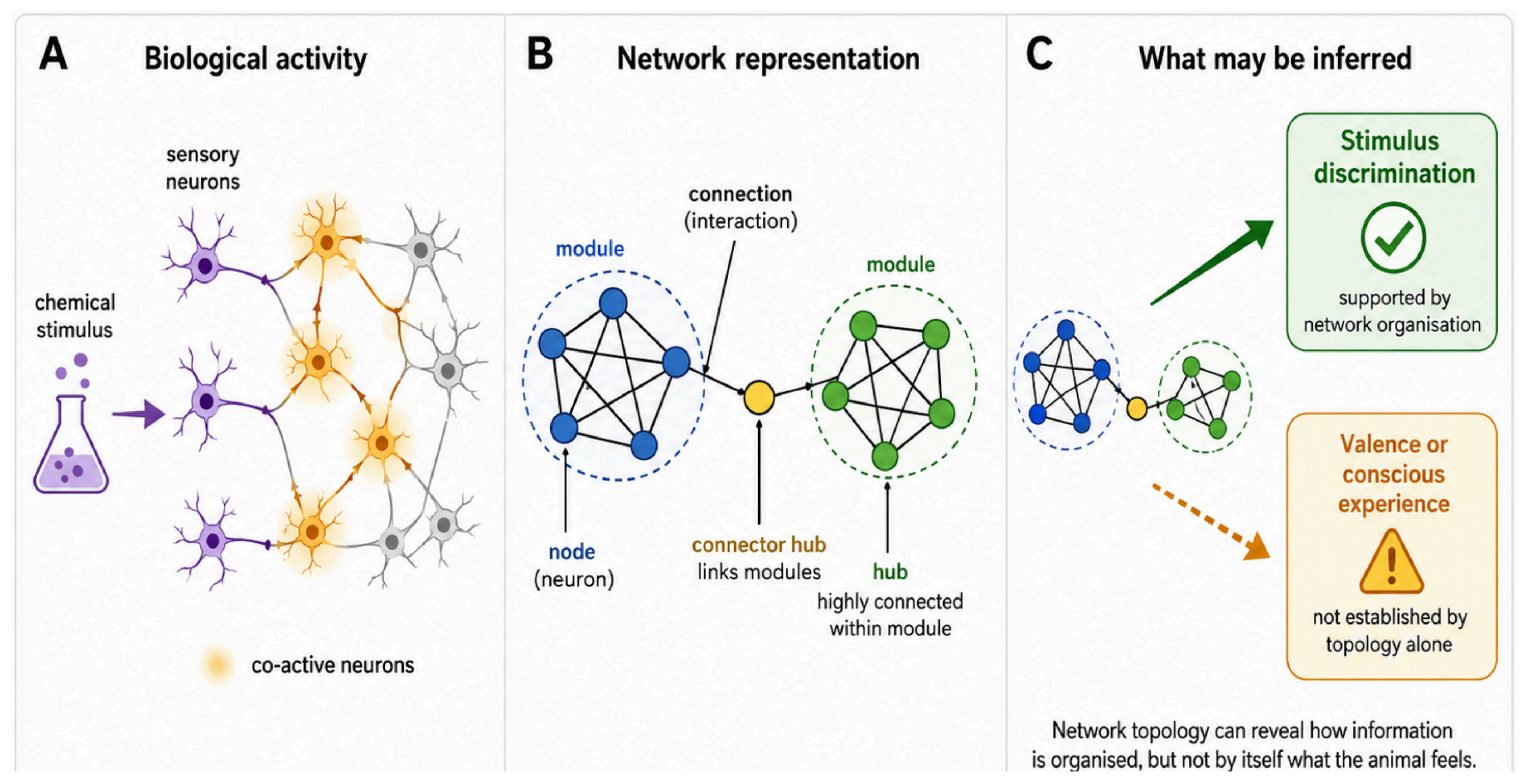
From neural activity to network topology—and its inferential limits. (**A**) A biological stimulus can activate partly overlapping groups of neurons. (**B**) Their interactions can be represented as nodes and connections, revealing modules and hubs. (**C**) This organisation may support inferences about stimulus discrimination and neural integration, but it does not by itself establish stimulus valence or conscious experience.

Network analysis can describe the organisation of neural activity but does not by itself establish subjective experience. Graph analysis represents neurons or brain regions as nodes and structural or functional relationships as edges [68]. Functional relationships inferred from activity do not necessarily correspond to direct anatomical connections. These analyses can reveal stimulus-related organisation, but their interpretation depends on how the network is constructed. A network pattern therefore requires independent validation before it can be interpreted as an indicator of valence or consciousness (Figure 2).

In *Caenorhabditis elegans*, features of functional neural interactions helped researchers identify which chemosensory stimulus had been presented. However, the tested features did not reveal a common neural pattern distinguishing attractive from repulsive stimuli [69]. This illustrates the distinction between identifying a stimulus and establishing its biological value. Methodological limitations may have obscured a shared representation of valence, so its absence cannot be inferred from these findings.

Activity across groups of neurons can reveal coordinated patterns associated with internal states that are not apparent when individual neurons are examined alone. Nair, Linderman and Anderson review how coordinated patterns of activity across hypothalamic neurons may represent internal states and how the tendency of these patterns to remain stable or recur may help explain why such states persist [70]. Identifying such dynamics does not, by itself, establish the valence or conscious experience of the represented state. In mice exposed to another mouse acting as an intruder, disrupting both oxytocin and vasopressin receptors in adult hypothalamic VMHvl *Esr1*-positive neurons reduced attack behaviour and the persistence of neural activity. More specifically, it abolished a coordinated activity pattern capable of maintaining different levels of activation over time, termed a line attractor. The overall neural activity and responses to the intruder’s sex or behaviour changed only modestly [71]. This suggests that neuropeptide signalling helps sustain an aggression-related neural state while largely preserving responses to social cues. The study did not establish whether this state was consciously felt or supported flexible choice between competing actions.

Recurrent integration is treated here as a candidate mechanism for maintaining and flexibly using information, not as an established universal prerequisite for affective experience. Findings about conscious perceptual access require additional justification before they are applied to basic affective processes [52,57].

### 5.5. Comparative Neural Architectures and Dimensions of Consciousness

Comparative neuroanatomy alone cannot identify a definitive evolutionary boundary for affective consciousness [65]. Birch et al. propose five dimensions for comparing animal consciousness: perceptual richness, evaluative richness, unity of experience at a given moment, integration across time, and self-consciousness [66]. These dimensions describe potentially different profiles rather than a single ascending scale. We use them to organise empirical findings and identify unanswered questions about animal experience. In this review, they provide a conceptual framework rather than a validated scale for measuring subjective experience. Within our framework, we propose functional links between these dimensions and the processes discussed above. Differentiated sensory processing may contribute to perceptual richness; valuation and action-selection mechanisms may contribute to evaluative richness; bidirectional signalling between brain regions may help integrate sensory and internal information into a unified experience; and persistent states and memory may contribute to integration over time. These are hypotheses for comparative assessment. We distinguish the possibility of feeling an unpleasant state from the ability to reflect on oneself as experiencing it. Our framework therefore leaves open the possibility of negatively valenced experience in animals for which reflective self-consciousness has not been demonstrated.

Amphioxus illustrates the distinction between organised neural control and evidence of affective valuation. Lacalli and Candiani combined ultrastructural and neurotransmitter data to interpret circuits involved in fast and slow swimming in amphioxus larvae [72]. Their analysis links neural anatomy and neurotransmitter identity to the control of fast and slow swimming but leaves unresolved how the swimming rhythm is generated and how connections between the two systems function [72]. It did not test persistent valence, motivational trade-offs or the value of relief, so its findings cannot establish whether these are present or absent in amphioxus.

Zebrafish studies allow these questions to be examined separately: how noxious stimulation changes behaviour, how previous conditions influence later choices, and how neural mechanisms maintain a defensive state.

In zebrafish larvae five days after fertilisation, exposure to 0.1% acetic acid reduced the proportion of time spent swimming. This reduction was not detected in larvae pretreated by immersion in aspirin (2.5 mg/L), lidocaine (5 mg/L) or morphine (48 mg/L) [73]. This demonstrates pharmacological modulation of a behavioural response during a noxious challenge but does not establish a persistent state after exposure or the motivation to obtain relief.

In a judgement-bias task, zebrafish learned that one colour-marked arm of a maze led to a food reward, whereas another was associated with being chased by a net. Researchers then measured how quickly fish entered an arm with an intermediate location and colour cue, for which the outcome was uncertain. At four months, telomerase-deficient fish took longer to enter this ambiguous arm than age-matched wild-type fish, a response interpreted as a more pessimistic expectation. This difference was not detected in separate groups tested at nine months. Both genotypes responded similarly to the familiar rewarded and aversive arms, making a general difficulty in detecting cues or moving through the maze less likely [74].

These findings support an age-dependent alteration in responses to ambiguity; their interpretation as conscious negative affect remains indirect.

Zhao et al. exposed zebrafish to an alarm substance from other zebrafish. After an initial burst of erratic swimming, the fish remained largely stationary and then swam slowly while responding to weaker aversive stimuli than before exposure. Reduced swimming persisted even after transfer to fresh water. This vigilance state was accompanied by sustained synchronised activity in the dorsal pallium. Manipulations of serotonin signalling and its 5-HT7 receptors supported a causal role in generating and maintaining the state [75]. These findings demonstrate regulation of a persistent defensive state without independently establishing that it was experienced as unpleasant.

A separate study examined responses to intraperitoneal acetic acid in adult zebrafish. The fish showed abdominal writhing-like movements and repetitive circling near the top of the tank, accompanied by increased whole-body cortisol and darker pigmentation. Morphine prevented these changes, and naloxone antagonised its effects [76]. This supports opioid modulation of coordinated behavioural and physiological responses to a noxious challenge.

Together, the zebrafish studies discussed above address responses to noxious stimulation, evaluation of ambiguous cues and regulation of persistent internal states. Because these findings come from different experiments and conditions, they do not establish that all these processes form a single persistent, consciously negative state.

Birds and mammals illustrate how different forebrain organisations can share connectional principles. Shanahan et al. reconstructed a pigeon forebrain connectivity matrix and identified modular, small-world organisation with highly connected hubs [77]. Stacho et al. used fibre imaging and tract tracing to identify repeated, column-like circuits and reciprocal connections in the sensory pallium of pigeons and barn owls, including connections to associative and motor regions [78]. These findings provide anatomical support for integration in an organisation that differs from the mammalian neocortex. They do not directly establish equivalent affective experience. The question posed by our framework is whether different architectures link sensory and internal information to valuation, memory and behavioural selection (Figure 2).

The octopus provides a comparison beyond vertebrate architecture. Following acetic acid injection into an arm, octopuses showed injection-site grooming and learned to avoid the associated chamber. Local lidocaine treatment produced preference for its associated chamber in acid-injected animals but not in saline-injected controls [79]. This finding is consistent with pain relief having a rewarding effect. However, Schnell et al. [80] identify an important limitation: the experiment lacked an acid-injected group receiving the same conditioning sequence with saline replacing lidocaine. Without that control, it remains uncertain whether the preference specifically reflected the value of pain relief.

In a separate reduced preparation with the central brain removed, brachial-connective recordings showed ongoing activity lasting more than 30 min after acetic acid injection. Local lidocaine rapidly silenced this activity and suppressed enhanced mechanically evoked responses [79]. These recordings demonstrate sustained nociceptive signalling, rather than conscious processing within the brain. Taken together, the findings support learned avoidance and local nociceptive blockade and are consistent with an affective-pain interpretation. They do not directly establish subjective feeling or explain how signals from the arms are integrated centrally.

Schnell et al. also show why evidence should be assessed separately across cephalopod groups. Their review finds strong support for sentience in octopuses and cuttlefish but substantial gaps in less-studied groups, particularly nautiluses. Differences in evidential support should not be treated as a ranking of subjective experience [80].

We therefore consider possible differences in the form of conscious experience without placing species on a single evolutionary scale. Where negatively valenced experience occurs, its character may depend on perceptual detail, evaluative differentiation, simultaneous integration, temporal persistence and self-related processing. This proposal does not permit direct comparison of subjective intensity between species. It identifies the distinct claims that behavioural and neurophysiological evidence must address.

### 5.6. Persistence and Consequences for the Whole Organism

Within this framework, a negatively valenced experience may be welfare-relevant because it is acutely severe, persists or recurs. Persistence and impaired recovery can provide additional evidence of sustained burden, but neither is required for an acute experience to be seriously adverse. Conversely, persistent physiological disturbance does not by itself establish experienced suffering.

Glial mechanisms contribute to the regulation and maintenance of neural function. Microglia participate in synaptic remodelling [81], while astrocyte–microglia interactions contribute to homeostasis and responses to injury [82]. Neuroglia have diversified during nervous-system evolution [83]. Myelin supports rapid axonal conduction and neuronal maintenance [84], and astrocytes can modulate circuit activity and network states [85]. Enteric circuits regulate gastrointestinal motility, secretion and homeostasis [86]. The blood–brain barrier also acts as an endocrine interface between the circulation and the brain [87,88]. These findings identify mechanisms through which neural and bodily functions are regulated; they do not establish that a particular adverse event produces a persistent negatively valenced state. Safron proposes that cognition and action depend on interactions between the brain, body and environment, in which an organism updates its predictions from sensory information and acts to regulate its bodily and environmental conditions [89]. This is an explanatory proposal, rather than independent evidence of conscious experience.

The empirical question is whether changes continue beyond the initiating event, affect normal functioning, and change following relief or recovery. Within our framework, evidence of negative valuation should be considered alongside changes in sleep, feeding, social interaction or physiological regulation. Their timing and response to intervention help distinguish sustained consequences from an immediate response. An ongoing injury must also be distinguished from a state that persists after its initiating condition has been removed. Mendl and colleagues propose that longer-term mood reflects accumulated emotional events and influences subsequent responses to ambiguous situations [54]. This is a conceptual account, not a validated measure of conscious suffering.

A mouse study examined how an ongoing nerve injury affected both relief-related preferences and sleep. Following partial sciatic nerve ligation, sleep recordings on postoperative day 11 showed sleep fragmentation and reduced REM sleep. Conditioned place preference for gabapentin was assessed on days 11–15, providing evidence consistent with rewarding relief from an ongoing adverse condition. CRISPR/Cas9-mediated reduction in kappa opioid receptor expression in the anterior cingulate cortex, performed before nerve ligation, prevented gabapentin-associated place preference, normalised sleep fragmentation and partially restored REM sleep, while mechanical allodynia remained [90]. These findings support the contribution of cortical kappa opioid signalling to relief-related valuation and sleep disturbance following nerve injury. Because the intervention preceded injury, they do not demonstrate reversal of an established state or recovery after removal of the underlying condition. Conscious suffering remains indirectly inferred.

Welfare frameworks emphasise both the reduction in negative experiences and opportunities for positive engagement [91,92]. For the present framework, assessment should therefore consider the intensity, duration and recurrence of the inferred negative state, together with controllability, normal activity and recovery. These are assessment criteria, not a validated formula for measuring subjective suffering. Persistent functional impairment strengthens a welfare inference when it converges with evidence of negative valuation; neither impairment nor an aversive stimulus alone establishes chronic suffering.

Comparative assessment therefore draws on converging evidence rather than a single brain structure, transmitter or network marker. Neuromodulatory, glial, autonomic and network findings can help explain how sustained effects are generated and maintained. Evidence of negative valuation, functional consequences and responses to intervention addresses distinct parts of the inference. Their convergence can support an interpretation of sustained affective burden without providing direct access to the animal’s experience.

## 6. An Operational Framework for Neurobiological Plausibility

Table 2 distinguishes the empirical questions involved in inferring welfare-relevant internal states. These questions are not successive levels of consciousness, and evidence addressing one does not automatically resolve the others. The proposed evidence conditions guide qualitative appraisal; they are not validated diagnostic thresholds. The framework integrates findings from different studies and species, but the complete set of proposed links has not been tested as a single causal model.

Gross brain size or the relative volume of a region may contribute to comparison but cannot by itself quantify consciousness or emotional intensity. Network analysis provides complementary information about connectivity and the organisation of neural activity [68].

We treat inference about welfare-related internal states as a series of questions, not as a count of markers or an unvalidated numerical score. First, the assessor states what was observed and what internal state is being inferred. The assessor then considers alternative explanations; whether the animal could perceive the stimulus, choose between options and perform the response; whether each marker has the expected direction and has been validated in that species and setting; whether different findings constrain distinct alternative explanations; and whether interventions or changes over time distinguish between explanations. Each conclusion is restricted to the inference question and biological process supported by the evidence. A study may address several questions, but support for one does not automatically establish the others. The level of welfare concern is reported separately from confidence in that conclusion.

When findings conflict, we first ask whether they concern the same state, time period and experimental conditions. We then consider measurement validity, biological specificity and whether controlled interventions distinguish competing explanations. Correlated measures are not counted as independent support. A negative finding is informative only when the study could reasonably have detected the proposed effect. If the conflict remains unresolved, we retain uncertainty for that claim rather than assigning the animal to a higher or lower level of consciousness.

The framework distinguishes capacity, occurrence and welfare significance and assesses confidence in each conclusion. Capacity concerns whether the species’ biological organisation supports the possibility of valenced experience. Occurrence concerns whether the evidence supports such an experience in this individual under the conditions examined. Welfare significance concerns the inferred experience’s severity, duration, recurrence, controllability and consequences for normal functioning. These questions require distinct justification. Evidence of functional impairment can inform welfare assessment but does not independently establish consciously experienced suffering.

Studies using more than one endpoint illustrate why different questions must remain separate. Rault et al. investigated nitrous oxide (N_2_O) as a potentially less aversive alternative to carbon dioxide (CO_2_) for piglet euthanasia. Free-choice approach–avoidance tests assessed behavioural aversion to gas exposure. In a separate experiment, Electroencephalography (EEG) recordings measured changes in cerebral electrical activity in piglets already maintained under anaesthesia [93]. These changes included the onset of an isoelectric EEG—a flat trace with no detectable electrical activity in the recording. Such a trace does not establish that every neuron has stopped functioning.

The welfare question concerns both how unpleasant the exposure is while an animal remains conscious and how long that experience lasts. Behavioural avoidance provides evidence of aversion, while the EEG experiment demonstrates cerebral suppression under the tested anaesthetic conditions. Because the piglets were already anaesthetised, these recordings did not directly establish when an initially awake animal would lose consciousness. The experiments therefore cannot determine the duration of consciously experienced aversion during gas exposure. More generally, an EEG criterion used to infer unconsciousness requires independent support; applying that criterion does not itself validate the inference.

This approach preserves the relevance of behavioural and neural evidence while keeping its interpretation proportionate to what was measured. Inability to measure subjective experience directly does not establish its absence, and behavioural resemblance alone does not establish that an animal’s experience is equivalent to a human one.

Table 3 summarises the appraisal rules and safeguards used to keep each conclusion proportionate to the evidence.

These appraisal rules also apply to artificial systems. The following comparison examines which functions can be implemented computationally and which further inferences about experience remain unresolved.

## 7. What Artificial Systems Reveal and What They Do Not

Comparing artificial and biological systems helps distinguish the ability to perform a function from evidence that the function is consciously experienced. It also allows us to ask which biological properties contribute to that function and whether they matter for experience. Artificial networks can implement direct-fit learning, recurrent processing, functional modules and diffusion-based modulation [8,39,40]. In a different kind of comparison, living neural cultures coupled to a task through feedback showed adaptive changes in network activity and performance [94]. These cultures are biological systems embedded in a technical environment. Both kinds of comparison inform questions about learning and organisation while leaving subjective experience unresolved.

A computational account can describe what a system does without resolving whether its processing is consciously experienced [95]. Implementing diffusion, recurrence, learning from feedback, internal representations or higher-level control therefore does not by itself establish feeling. Equally, artificial implementation does not show that these processes are unnecessary in a conscious animal. A preprint proposing more autonomous AI learning through observation and action illustrates this distinction: it concerns the development of functional capacities, rather than a demonstration that outcomes are experienced as pleasant or unpleasant [96].

Functional comparisons can nevertheless contribute to an assessment of consciousness when their theoretical assumptions are explicit. Butlin and colleagues propose using indicators derived from scientific theories to assess the likelihood of consciousness in AI systems [97]. The significance of an indicator depends on the theory from which it is drawn and on whether that theory applies to the system being assessed. Meeting proposed indicators therefore provides theory-dependent evidence, rather than an independently validated demonstration of consciousness. We accordingly treat artificial systems as comparison cases whose subjective status remains unresolved.

A recent computational example concerns the availability of information to different processes within a system. In a preprint, Gurnee et al. used the Jacobian lens to identify workspace-like representations in large language models [98]. Interventions on these representations altered verbal reports and flexible internal reasoning, while the tested routine processes were relatively preserved. These findings resemble some functional properties associated with conscious access. However, the observed broadcasting occurs as information passes forward through successive model layers; it does not demonstrate the recurrent interactions described in biological workspace accounts. The method also captures only part of the representational structure, principally through token-linked concepts. Failure to detect a representation therefore does not establish that the corresponding processing is absent. The study provides a comparison of functional organisation, rather than a validated measure of subjective experience across systems or taxa.

Other proposals focus on the regulation of needs rather than the availability of information. Solms et al. describe an artificial-agent architecture and propose hedonic place preference as a possible test of affective consciousness [99]. Their account predicts how such behaviour could arise; it does not report a completed experimental demonstration of artificial hedonic place preference. The proposed mechanism concerns how an agent selects actions when it must regulate internal need variables using incomplete information about its own state and surroundings. The authors acknowledge that passing the behavioural test alone would not establish subjective experience. For our framework, the unresolved step is the inference from regulating represented needs and uncertainty to experiencing pleasure or uncertainty. Computational explanation alone neither establishes nor refutes that inference. In animals, place preference likewise requires interpretation alongside the biological condition, experimental controls and other relevant findings.

Candia-Rivera’s interoception-inspired framework develops a related proposal by combining regulation of internal states, anticipation of future needs and active information gathering [100]. It translates principles drawn from biological regulation into possible computational architectures. This creates opportunities to investigate how internal-state regulation affects learning and action while preserving the distinction between regulating a need variable and experiencing that need.

The same distinction helps clarify different meanings of autonomy. A cleaning robot can detect obstacles, map a room, monitor its battery and return to a charger. These functions allow it to carry out a task and maintain access to power. Biological autonomy involves a broader organisation in which metabolism, repair, development and bodily regulation maintain a living organism [101,102]. Comparing these forms of self-maintenance identifies differences in how systems sustain their activity. It does not, by itself, determine which systems can have subjective experience.

Biological neurons participate in this living organisation: they help regulate the body, and their activity is shaped by its changing needs. Their processing depends on dendritic activity, ion channels, intracellular signalling, chemical and electrical synapses, metabolic support and interactions with glial cells [81,82,83,84,85,103,104,105,106,107,108]. Neural circuits also develop within living tissues, as illustrated by accounts of the enteric nervous system and *Xenopus* spinal cord [105,106,107]. These properties identify mechanisms whose contributions can be investigated experimentally. Their presence alone does not establish consciousness, just as reproducing selected functions in an artificial network does not establish equivalent biological organisation. Similar performance may arise through different mechanisms; functional resemblance therefore does not establish shared evolutionary origins or equivalent implementation.

Klatzmann and Doerig sharpen this distinction by separating the claim that biology is intrinsically necessary for consciousness from the proposal that particular biological properties enable relevant forms of information processing [109]. They argue that the latter allows empirical investigation of what those properties contribute. We accordingly treat metabolism, vulnerability and self-maintenance as properties whose roles require investigation. Artificial models can help identify alternative explanations for observed functions, while biological experiments can test the contribution of particular mechanisms. Neither functional similarity nor biological difference alone settles whether conscious experience is present. The comparison therefore returns us to the animal: what evidence links an observed function to valuation and possible experience within its biological organisation? Section 8 applies this question to mammals and birds.

## 8. Species-Specific Inference in Mammals and Birds

A comparative framework should not use the human brain or human expression as its sole template. Animal consciousness may vary along several dimensions rather than across one threshold [66]. Neural correlates established in humans remain informative, but reverse inference weakens as species differ in anatomy, sensory ecology and behaviour [65,110,111]. Similar functions may be produced by different architectures, and similar internal states may be expressed differently across species. Comparative cell atlases reveal both conserved and divergent neuronal types in bird and mammal pallia, cautioning against a direct correspondence between their regions [112].

Mammals and birds are a useful comparison because both possess integrated arousal, motivational, nociceptive and neuromodulatory systems, together with pallial networks that support flexible learning. They achieve these functions through different anatomical arrangements.

Our framework therefore does not use the mammalian neocortex as its anatomical standard. Instead, it asks whether sensory and internal information is linked to valuation, recurrent integration, flexible behaviour and regulation of the whole organism.

Farm-animal cognition shows that flexible use of information is not confined to primates. Cattle, horses, pigs and small ruminants can recognise and remember individuals, use social information and, in some cases, respond to human attention, direct attention to inaccessible resources and solve novel physical tasks [113]. These abilities show that information can be retained, combined with context and redirected towards alternative actions. They are not direct proof of conscious feeling; their relevance depends on links with valuation, persistence and effects on the whole animal.

Behavioural tests must also be interpreted according to the construct they were designed to assess. In growing pigs, open-field responses changed with pharmacological treatment, social companionship and previous exposure. Multivariate analysis separated the form of the response from its magnitude or arousal [114]. The same outward measure may therefore change through fear, social buffering, habituation or exploration and cannot be assigned one fixed affective meaning.

Ecology further changes the meaning of outward signs. Mammalian brain regions scale under developmental, metabolic and ecological constraints, and the idea of one uniform ‘limbic system’ can obscure this diversity [115]. Predators have specialised circuits for search, pursuit and capture [116]. Prey species such as horses continually monitor their surroundings for potential threats, mobilise rapidly in response and benefit from social buffering. They may also suppress obvious signs of pain or vulnerability [117,118]. Equine sensory organisation favours vigilance and rapid responses to uncertainty [119]. Species-specific interpretation is therefore essential: the same outward response may have different causes, and the absence of an obvious response may be uninformative. These differences matter when evidence is used to evaluate housing, management and human use.

## 9. Implications for Domestic Animal Welfare and Human Use

The framework does not decide in advance whether animal use is acceptable. It distinguishes limited, recoverable challenge from severe, recurrent or persistent negative states that impose cumulative burden. Welfare is not secured merely by preventing injury, disease or overt distress. Housing and management also influence predictability, control, social continuity and opportunities for exploration, learning and species-appropriate engagement [113].

The Five Domains Model uses a related two-step approach. In free-roaming horses, investigators first linked measurable physical and functional signs to their biological effects and then cautiously inferred possible mental experiences [120]. This makes the step from observation to interpretation explicit and requires support from physiology, neurophysiology, neuroethology and affective neuroscience. Our framework further separates four questions: whether the species has the relevant capacity, whether the state is occurring now, what acute or sustained welfare burden it may impose, and how confident we can be in these. Neither framework should treat numerical scores as a universal scale of subjective intensity.

Manageable cognitive challenge, stable social relationships and positive human–animal interactions may support competence and positive affect. Task performance alone, however, does not prove positive experience. That interpretation requires supporting evidence from motivation, voluntary participation, physiological regulation, persistence and later behaviour.

## 10. AI-Assisted Observation and the Continuing Need for Biological Validation

**Figure 3:**
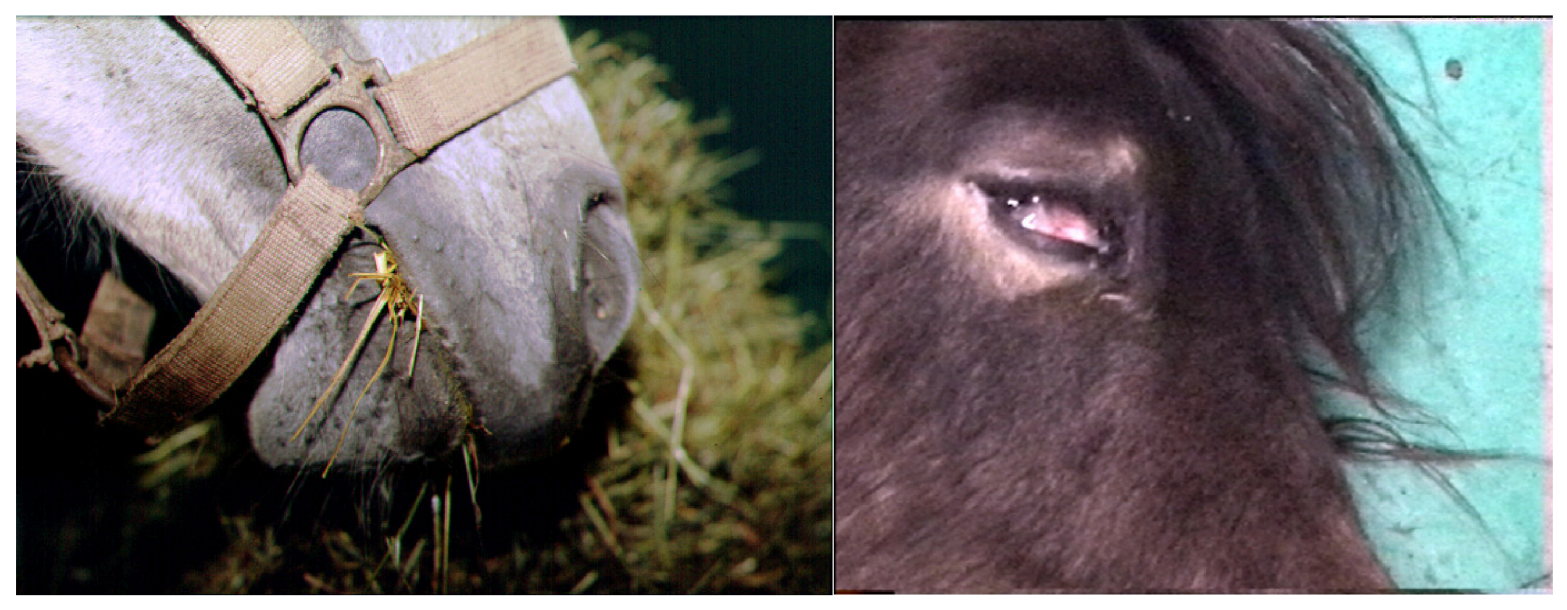
Facial expression is useful evidence only in clinical and biological context. The images show trismus (**left**) and protrusion of the third eyelid caused by retractor bulbi muscle spasm (**right**) in horses with tetanus. Although these features may resemble pain-associated expressions, they primarily reflect disease-specific neuromuscular dysfunction and should not be read as a typical equine pain face (Source: Equine Unit, University of Veterinary Medicine Vienna).

Applying these principles requires observations across time and under the conditions in which animals actually live. AI can help collect and analyse such observations, but its classifications require the same biological scrutiny as human judgements. It can measure gait asymmetry, facial movements, posture, vocalisations and activity budgets and combine several data sources. Automated analysis of equine facial expression illustrates both the benefit and the limitation: a model identifies a facial pattern associated with pain; it does not measure pain itself [121]. Figure 3 illustrates why facial signs require interpretation in their clinical context.

Validation studies make the distinction concrete. A classifier based on horse and mouse facial action units separated pain-labelled from control conditions with an accuracy above 70%. This is a useful starting point for automated recognition but not a direct measurement of felt pain [122]. Composite equine pain scales have likewise shown variable reproducibility, weak sensitivity and only minimally acceptable internal consistency [123]. Discrimination, agreement between observers and responsiveness to surgery or analgesia are valuable properties, but they do not show that equal score intervals represent equal differences in subjective intensity.

In socially experienced domestic animals, behaviour may also reflect recognition of particular handlers, learned expectations and sensitivity to human attention [113]. Automated classification of gaze, approach, responsiveness or facial movement should therefore distinguish, as far as possible, immediate state from learning and relationship history.

Circular validation is the main risk. Human observers define a category, select examples, label the data and train a model to reproduce those labels. The model’s output may then be presented as independent confirmation of the original interpretation. Welfare scientists should therefore take part in the design and biological validation of AI tools; technical accuracy is not the same as biological validity [124]. Professional judgement is not immune to this problem. In a survey of conservation professionals, views of animal consciousness varied mainly with the animal’s taxonomic group and the respondent’s education [125]. Broomé and colleagues identify related problems in reference-label construction and evaluation of generalisation in computer-vision-based recognition of animal pain and emotion [126].

Modern machine-learning studies face another important problem: data leakage. Related observations in the training and test sets can make performance appear better than it will be on independent data. For example, when individual images or video frames are split at random, a model may recognise the animal, background or equipment instead of a welfare-related sign. Data should therefore be separated at the level relevant to the intended application, such as animal, group, farm, clinic or study [126,127,128].

Riaz et al. [129] illustrate why this choice matters. Using accelerometer data from 383 dairy cows on 11 farms, they evaluated models against veterinary-diagnosed severe foot lesions. The researchers compared two ways of testing the models: using cows from farms already represented in the training data or using cows from farms the models had not encountered during training. In the second approach, performance varied more between farms, and the agreement between predicted probabilities and observed lesion frequencies also differed. These findings support evaluating models under conditions that reflect their intended use. Detecting a lesion, however, does not directly measure the animal’s experienced pain.

External validation should use animals, settings and recording conditions that were not part of model development. Studies should report discrimination, calibration, sensitivity and specificity, including performance when class frequencies are unequal [128,130]. Researchers should also test for irrelevant shortcuts, such as restraint equipment, treatment context, observer behaviour or location [131]. Because the overall results may conceal poor performance in particular breeds, ages, diseases or management systems, evaluation should address relevant subgroups, uncertainty and differences between development data and the intended setting [127,128,130,131].

Decision thresholds should reflect the relative consequences of false-positive and false-negative classifications rather than being chosen solely to maximise predictive accuracy [132]. The model should define the setting in which it has been validated and withhold a prediction when the input is incomplete or outside that setting [133]. For welfare-related decision support, predictive performance, generalisability, uncertainty and the biological consequences of classification errors should therefore be considered together.

**Figure 4:**
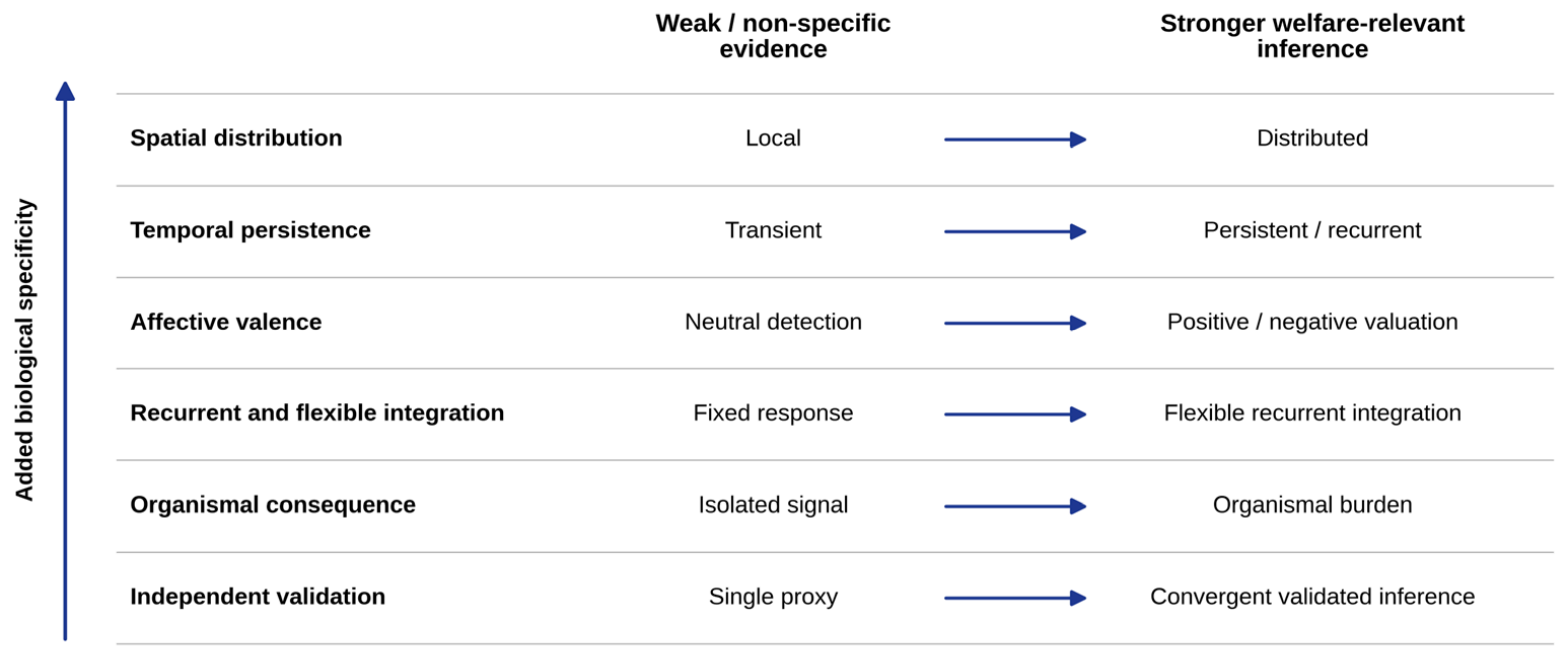
Dimensions of internal-state inference from neural event to welfare-relevant organismal burden. A welfare-relevant inference becomes stronger when evidence converges across multiple dimensions: spatial distribution, temporal persistence, affective valence, recurrent and flexible integration, organismal consequence and independent validation. No single marker is sufficient. External output remains evidence to be interpreted, not direct access to subjective experience.

Agreement with expert labels for pain, fear or distress shows that a model can reproduce those labels. It does not validate the claimed subjective state. Biological validation therefore needs evidence from outside the labelling process. Relevant evidence may include clinical findings, physiology, response to analgesics or other targeted treatment, recovery over time, species-specific behaviour, or outcomes predicted by the proposed interpretation. This matters because one outward sign can arise from several internal states, while the same internal state can produce different signs [92,120]. AI should therefore support observation and hypothesis testing, not act as an independent authority on sentience or suffering. Figure 4 summarises this reasoning.

## 11. Conclusions

Assessing animal suffering requires distinguishing what an animal does, what biological state the evidence supports, and whether that state is plausibly experienced as seriously adverse. This review separates the animal’s capacity for experience, the occurrence of a particular state and its welfare significance while assessing confidence in each inference. Its contribution is to connect these questions with evidence about stimulus detection, wider neural modulation, valuation, flexible integration and consequences for the whole organism. These processes organise the assessment; they do not constitute a validated causal sequence or a scale of consciousness.

Complementary evidence strengthens an inference when it constrains different plausible explanations. Behavioural preferences and motivational trade-offs can inform valuation; neural and pharmacological findings can clarify mechanisms; and observations over time can establish persistence, recurrence and responses to intervention. Their contribution depends on the species, experimental conditions and validity of the measures. Several correlated signs of the same response should not be counted as independent confirmation of feeling.

Artificial systems help clarify the limits of functional evidence. Learning, recurrence, diffusion-like modulation and adaptive control can be implemented without independently establishing subjective experience. This does not show that these functions are unnecessary in conscious animals or that artificial systems are necessarily non-conscious. Likewise, the biological properties of neurons and living organisms identify mechanisms for investigation rather than sufficient markers of feeling.

Comparative evidence supports examining different forms of neural organisation without placing species on a single evolutionary ladder. Birch et al.’s dimensions provide a framework for asking how perception, evaluation, unity of experience, integration across time and self-related processing may differ [66]. The studies considered here address aspects of the mechanisms that could contribute to these dimensions; they do not directly measure complete experiential profiles or permit comparison of subjective intensity between species.

For welfare assessment, severity, duration, recurrence, controllability and recovery help characterise the burden of an inferred negative experience. A brief experience may be seriously adverse, while persistent physiological disturbance does not by itself establish suffering. Uncertainty about subjective experience should therefore be reported separately from the level of welfare concern. Assessment should also consider opportunities for positive engagement, alongside the prevention and relief of adverse states.

In livestock production, companionship, work and equestrian sport, this approach directs attention to what animals experience under particular conditions and what can be changed to improve their welfare. AI can extend observation and reveal patterns over time, but agreement with human labels is not independent biological validation. Its practical value depends on whether its outputs support sound interpretation and useful decisions for the animals being assessed.

## Figures and Tables

**Table 1:** Operational definitions of key terms used in this review.

Term	Use in This Review
External output	Observable behaviour, performance, physiological signal or AI-generated classification. It is evidence, not direct access to internal state.
Internal state	A biologically organised state within the organism that may include neural, autonomic, endocrine, metabolic and behavioural components.
Affect	A positively or negatively valenced state that influences motivation, learning and action, without assuming that it is consciously felt.
Conscious affect/feeling	The possible subjective component of an affective state. It cannot be inferred from one marker alone.
Valence	The positive or negative biological value of a state for the organism.
Arousal	Mobilisation or activation of the organism. It is not by itself a welfare conclusion; its meaning depends on valence, context, controllability, persistence and recovery.
Nociception	Detection and neural processing of potentially damaging stimuli. It does not automatically equal pain.
Pain	A negative state plausibly linked to actual or potential tissue damage, requiring central integration beyond nociceptive input alone.
Suffering:	Consciously experienced negative affect that is seriously adverse because of its intensity, duration, recurrence or other burdensome features. Its occurrence is inferred indirectly from converging evidence.
Organismal burden:	Acute or sustained adverse consequences for the animal’s functioning, bodily regulation, behaviour, health or recovery; these consequences do not independently establish subjective suffering.
Proxy/marker	An indirect indicator of a possible internal state. No single proxy is treated as sufficient.
Convergent inference	Strengthening an interpretation when complementary findings constrain different plausible alternative explanations, rather than merely repeating correlated measures of the same response.
Operational autonomy	Capacity of an artificial system to act adaptively within externally designed, powered and maintained conditions.
Biological autonomy	Self-maintaining organisation of a living organism involving metabolism, development, repair, vulnerability and need.
Functional comparison case	An artificial or formal system used to compare functional organisation and alternative explanations, without presuming the presence or absence of subjective experience.

**Table 2:** Empirical questions, evidence conditions and limits in internal-state inference.

Inference Question	Proposed Evidence Condition	Supported Conclusion	Remaining Limitation
Stimulus detection	A reproducible stimulus-linked response, with relevant sensory and motor controls.	The stimulus was detected and processed.	Valence and experience are not established.
Distributed state modulation	Changes in neural activity or responsiveness beyond the immediate sensory-motor response, with evidence addressing their distribution, duration or causal mechanism.	Modulation extending beyond the immediate response is supported, within the measured systems and timescale.	Distribution and persistence require separate evidence; neither establishes valence or conscious experience.
Biological valuation	State-dependent choice, motivational trade-offs, effort or preference for relief, with relevant sensory, motor, learning and treatment confounds addressed.	Positive or negative biological valuation is supported for the tested condition.	Biological valuation does not independently establish conscious feeling.
Flexible integration	Retained information guides context-dependent or alternative actions; neural evidence addresses the proposed mechanism.	Information is maintained and flexibly used.	Flexibility alone does not establish consciousness; behavioural evidence alone does not demonstrate recurrence.
Welfare-relevant burden	Evidence of negative valuation considered alongside acute severity, duration, recurrence, controllability, functional consequences and recovery, as relevant to the condition.	The findings support an adverse-state interpretation and characterise its measured consequences.	Functional impairment alone does not establish suffering; subjective intensity remains indirectly assessed.

**Table 3:** Rules for qualitative integration of welfare-relevant evidence.

Rule	Question	Safeguard
Observation	What was directly seen or measured?	Keeps measurement separate from attributed internal state.
Alternatives	What else could produce the finding?	Prevents one sign from naming its own cause.
Opportunity	Could the animal perceive, choose and perform the response?	Limits inference from absent or constrained behaviour.
Directionality and transfer	Is presence and/or absence informative, and is meaning validated in this taxon?	Prevents one-way markers and cross-species assumptions from being overread.
Dependency	Do evidence streams constrain different alternatives?	Prevents correlated outputs from being counted as independent convergence.
Causal and temporal evidence	What changes after a targeted intervention or across onset, persistence, recurrence and recovery?	Temporal observations establish sequence and duration; controlled interventions can help distinguish causal explanations.
Claim limit	Which inference question and biological process does the finding support?	Prevents evidence for one process from being treated as proof of valence, conscious experience or suffering.
Concern and confidence	How important may the burden be, and how certain is that inference?	Prevents uncertainty from being equated with low concern.

## Data Availability

No new data were created or analysed in this study. Data sharing is not applicable to this article.

## Supplementary Materials

The following supporting information can be downloaded at: https://www.mdpi.com/article/10.3390/ani16213376/s1, Supplementary Material S1: Review Protocol and Epistemological Appraisal Framework, describing the search and appraisal procedures, the PubMed search strings and history from 22 July 2026, the supplementary Scopus coverage check, source-selection criteria, evidence-appraisal rules and the role of AI assistance; Table S1: Conceptual search templates, core concept blocks and representative publications; Table S2: Source disposition categories and their typical use; Table S3: Fields used for claim-level extraction and appraisal; Table S4: Appraisal questions and decision rules; Table S5: PubMed search intersections and retrieval counts; Table S6: Screening outcome of the records retrieved by the four-block PubMed search. The publications referred to in Supplementary Material S1 are cited in the main reference list [6,16,18,19,22,23,32,33,34,39,40,43,44,52,56,58,62,66,68,69,80,86,87,88,95,96,121,124,129].

## References

[1] European Union (2012). Consolidated version of the Treaty on the Functioning of the European Union. Off. J. Eur. Union..

[2] Mellor D.J. (2019). Welfare-aligned sentience: Enhanced capacities to experience, interact, anticipate, choose and survive. Animals.

[3] Dawkins M.S. (2017). Animal welfare with and without consciousness. J. Zool..

[4] Browning H., Veit W. (2023). Studying animal feelings: Integrating sentience research and welfare science. J. Conscious. Stud..

[5] Reimert I., Webb L.E., van Marwijk M.A., Bolhuis J.E. (2023). Review: Towards an integrated concept of animal welfare. Animal.

[6] Sneddon L.U., Elwood R.W., Adamo S.A., Leach M.C. (2014). Defining and assessing animal pain. Anim. Behav..

[7] Grinde B. (2024). Consciousness makes sense in the light of evolution. Neurosci. Biobehav. Rev..

[8] Hasson U., Nastase S.A., Goldstein A. (2020). Direct fit to nature: An evolutionary perspective on biological and artificial neural networks. Neuron.

[9] Bateson P. (1991). Assessment of pain in animals. Anim. Behav..

[10] Dawkins M.S. (2004). Using behaviour to assess animal welfare. Anim. Welf..

[11] Descovich K.A., Wathan J., Leach M.C., Buchanan-Smith H.M., Flecknell P., Farningham D., Vick S.-J. (2017). Facial expression: An under-utilised tool for the assessment of welfare in mammals. ALTEX.

[12] Arshavsky Y.I. (2026). On consciousness in animals and in artificial intelligence. J. Neurophysiol..

[13] Evangelista M.C., Monteiro B.P., Steagall P.V. (2022). Measurement properties of grimace scales for pain assessment in nonhuman mammals: A systematic review. Pain.

[14] Tomacheuski R.M., Monteiro B.P., Evangelista M.C., Luna S.P.L., Steagall P.V. (2023). Measurement properties of pain scoring instruments in farm animals: A systematic review using the COSMIN checklist. PLoS ONE.

[15] Cleeremans A., Mudrik L., Seth A.K. (2025). Consciousness science: Where are we, where are we going, and what if we get there?. Front. Sci..

[16] Mason G. (2026). Assessing laboratory animal welfare: The crucial importance of construct validity. Lab. Anim..

[17] Kirkeby-Hinrup A., Stephens A., Balogh Sjöstrand A., Overgaard M. (2025). Methodological issues in consciousness research: Theory comparison, the role of empirical evidence, and a replication crisis. Front. Psychol..

[18] Arendt D., Tosches M.A., Marlow H. (2016). From nerve net to nerve ring, nerve cord and brain: Evolution of the nervous system. Nat. Rev. Neurosci..

[19] Arendt D. (2021). Elementary nervous systems. Philos. Trans. R. Soc. Lond. B Biol. Sci..

[20] Levin M. (2014). Molecular bioelectricity: How endogenous voltage potentials control cell behavior and instruct pattern regulation in vivo. Mol. Biol. Cell.

[21] Cervera J., Levin M., Mafe S. (2023). Bioelectricity of non-excitable cells and multicellular pattern memories: Biophysical modeling. Phys. Rep..

[22] Moran Y., Barzilai M.G., Liebeskind B.J., Zakon H.H. (2015). Evolution of voltage-gated ion channels at the emergence of Metazoa. J. Exp. Biol..

[23] Arendt D. (2020). The evolutionary assembly of neuronal machinery. Curr. Biol..

[24] Elphick M.R., Mirabeau O., Larhammar D. (2018). Evolution of neuropeptide signalling systems. J. Exp. Biol..

[25] Gründer S., Assmann M. (2015). Peptide-gated ion channels and the simple nervous system of Hydra. J. Exp. Biol..

[26] Tessler M., Neumann J.S., Kamm K., Osigus H.-J., Eshel G., Narechania A., Burns J.A., DeSalle R., Schierwater B. (2022). Phylogenomics and the first higher taxonomy of Placozoa, an ancient and enigmatic animal phylum. Front. Ecol. Evol..

[27] Holland L.Z. (2015). The origin and evolution of chordate nervous systems. Philos. Trans. R. Soc. Lond. B Biol. Sci..

[28] Gusev E., Sarapultsev A., Komelkova M. (2025). Evolutionary trajectories of consciousness: From biological foundations to technological horizons. Brain Sci..

[29] Godfrey-Smith P. (2020). Metazoa: Animal Life and the Birth of the Mind.

[30] Aboitiz F. (2024). A History of Bodies, Brains, and Minds: The Evolution of Life and Consciousness.

[31] Zhang Z., Zhang S., Xu Y. (2025). The evolution of cognitive abilities in marine animals: A hypothesis based on insights about cognition gene polymorphisms in coelacanths and lungfish. Acad. Mol. Biol. Genom..

[32] Agnati L.F., Leo G., Zanardi A., Genedani S., Rivera A., Fuxe K., Guidolin D. (2006). Volume transmission and wiring transmission from cellular to molecular networks: History and perspectives. Acta Physiol..

[33] Gianni G., Pasqualetti M. (2023). Wiring and volume transmission: An overview of the dual modality for serotonin neurotransmission. ACS Chem. Neurosci..

[34] Hökfelt T., Barde S., Xu Z.Q.D., Kuteeva E., Rüegg J., Le Maitre E., Risling M., Kehr J., Ihnatko R., Theodorsson E. (2018). Neuropeptide and small transmitter coexistence: Fundamental studies and relevance to mental illness. Front. Neural Circuits..

[35] Nagy J.I., Pereda A.E., Rash J.E. (2018). Electrical synapses in mammalian CNS: Past eras, present focus and future directions. Biochim. Biophys. Acta Biomembr..

[36] Pérez-Fernández J., Barandela M., Jiménez-López C. (2021). The dopaminergic control of movement: Evolutionary considerations. Int. J. Mol. Sci..

[37] Leroy F., Gimenez P., Krabichler Q., Grinevich V. (2026). Evolution of neuropeptides: From diffusing molecules to modulators of synaptic transmission. Prog. Neurobiol..

[38] Merighi A., Sbriz M., Lossi L. (2026). Chemical multiplexing in the nervous system: Molecular architecture, functional stratification, and pathophysiological plasticity of neuropeptide-classical neurotransmitter cotransmission. Front. Mol. Neurosci..

[39] Velez R., Clune J. (2017). Diffusion-based neuromodulation can eliminate catastrophic forgetting in simple neural networks. PLoS ONE.

[40] Barretto-Bittar J., Levina A., Giannakakis E., Zeraati R. (2026). Diffusion of neuromodulators for temporal credit assignment. arXiv.

[41] Paz Suarez Araujo C., Fernandez Lopez P., Garcia Baez P., Moreno-Díaz R., Buchberger B., Luis Freire J. (2001). Towards a model of volume transmission in biological and artificial neural networks: A CAST approach. Computer Aided Systems Theory-EUROCAST 2001. EUROCAST 2001.

[42] Franz S., Heutehaus L., Tappe-Theodor A., Weidner N., Treede R.D., Schuh-Hofer S. (2023). Noxious radiant heat evokes bi-component nociceptive withdrawal reflexes in spinal cord injured humans—A clinical tool to study neuroplastic changes of spinal neural circuits. Front. Hum. Neurosci..

[43] Mashour G.A., Roelfsema P., Changeux J.-P., Dehaene S. (2020). Conscious processing and the global neuronal workspace hypothesis. Neuron.

[44] Sergent C., Corazzol M., Labouret G., Stockart F., Wexler M., King J.-R., Meyniel F., Pressnitzer D. (2021). Bifurcation in brain dynamics reveals a signature of conscious processing independent of report. Nat. Commun..

[45] Andersson L.S., Larhammar M., Memic F., Wootz H., Schwochow D., Rubin C.-J., Patra K., Arnason T., Wellbring L., Hjälm G. (2012). Mutations in DMRT3 affect locomotion in horses and spinal circuit function in mice. Nature.

[46] Grillner S. (2006). Biological pattern generation: The cellular and computational logic of networks in motion. Neuron.

[47] Tobin T. (1981). Drugs and the Performance Horse.

[48] Combie J., Shults T., Nugent E.C., Dougherty J., Tobin T. (1981). Pharmacology of narcotic analgesics in the horse: Selective blockade of narcotic-induced locomotor activity. Am. J. Vet. Res..

[49] Tobin T., Combie J., Shults T. (1979). Pharmacology review: Actions of central stimulant drugs in the horse. II. J. Equine Med. Surg..

[50] Schoeller F., Jain A., Pizzagalli D.A., Reggente N. (2024). The neurobiology of aesthetic chills: How bodily sensations shape emotional experiences. Cogn. Affect. Behav. Neurosci..

[51] Yokogawa T., Hannan M.C., Burgess H.A. (2012). The dorsal raphe modulates sensory responsiveness during arousal in zebrafish. J. Neurosci..

[52] Paul E.S., Sher S., Tamietto M., Winkielman P., Mendl M.T. (2020). Towards a comparative science of emotion: Affect and consciousness in humans and animals. Neurosci. Biobehav. Rev..

[53] Cabanac M. (1992). Pleasure: The common currency. J. Theor. Biol..

[54] Mendl M., Burman O.H.P., Paul E.S. (2010). An integrative and functional framework for the study of animal emotion and mood. Proc. R. Soc. B..

[55] Anderson D.J., Adolphs R. (2014). A framework for studying emotions across species. Cell.

[56] Brown S.A.B., Birch J. (2025). When and why are motivational trade-offs evidence of sentience?. Philos. Trans. R. Soc. Lond. B Biol. Sci..

[57] Panksepp J., Lane R.D., Solms M., Smith R. (2017). Reconciling cognitive and affective neuroscience perspectives on the brain basis of emotional experience. Neurosci. Biobehav. Rev..

[58] Quadt L., Critchley H., Nagai Y. (2022). Cognition, emotion, and the central autonomic network. Auton. Neurosci..

[59] Tovote P., Fadok J.P., Lüthi A. (2015). Neuronal circuits for fear and anxiety. Nat. Rev. Neurosci..

[60] Pessoa L. (2008). On the relationship between emotion and cognition. Nat. Rev. Neurosci..

[61] Ede T., von Keyserlingk M.A.G., Weary D.M. (2019). Assessing the affective component of pain, and the efficacy of pain control, using conditioned place aversion in calves. Biol. Lett..

[62] Ede T., Nogues E., von Keyserlingk M.A.G., Weary D.M. (2022). Pain in the hours following surgical and rubber ring castration in dairy calves: Evidence from conditioned place avoidance. JDS Commun..

[63] Block N. (1995). On a confusion about a function of consciousness. Behav. Brain Sci..

[64] Mudrik L., Faivre N., Koch C. (2014). Information integration without awareness. Trends Cogn. Sci..

[65] Birch J. (2021). The hatching of consciousness. Hist. Philos. Life Sci..

[66] Birch J., Schnell A.K., Clayton N.S. (2020). Dimensions of animal consciousness. Trends Cogn. Sci..

[67] Fesce R. (2023). Imagination: The dawn of consciousness: Fighting some misconceptions in the discussion about consciousness. Physiol. Behav..

[68] Papo D., Buldú J.M., Boccaletti S., Bullmore E.T. (2014). Complex network theory and the brain. Philos. Trans. R. Soc. Lond. B Biol. Sci..

[69] How J.J., Navlakha S., Chalasani S.H. (2021). Neural network features distinguish chemosensory stimuli in Caenorhabditis elegans. PLoS Comput. Biol..

[70] Nair A., Linderman S., Anderson D.J. (2026). The representation of affective internal states in the hypothalamus: A neural manifold view. Annu. Rev. Neurosci..

[71] Mountoufaris G., Nair A., Yang B., Kim D.W., Vinograd A., Kim S., Linderman S.W., Anderson D.J. (2024). A line attractor encoding a persistent internal state requires neuropeptide signaling. Cell.

[72] Lacalli T.C., Candiani S. (2017). Locomotory control in amphioxus larvae: New insights from neurotransmitter data. EvoDevo.

[73] Lopez-Luna J., Al-Jubouri Q., Al-Nuaimy W., Sneddon L.U. (2017). Reduction in activity by noxious chemical stimulation is ameliorated by immersion in analgesic drugs in zebrafish. J. Exp. Biol..

[74] Espigares F., Abad-Tortosa D., Varela S.A.M., Ferreira M.G., Oliveira R.F. (2021). Short telomeres drive pessimistic judgement bias in zebrafish. Biol. Lett..

[75] Zhao Y., Huang C.-X., Gu Y., Zhao Y., Ren W., Wang Y., Chen J., Guan N.N., Song J. (2024). Serotonergic modulation of vigilance states in zebrafish and mice. Nat. Commun..

[76] Costa F.V., Gonçalves F.L., Borba J.V., Sabadin G.R., Biasuz E., Santos L.W., Sneddon L.U., Kalueff A.V., Rosemberg D.B. (2023). Acetic acid-induced pain elicits stress and camouflage-related responses in zebrafish: Modulatory effects of opioidergic drugs on neurobehavioral phenotypes. Comp. Biochem. Physiol. C Toxicol. Pharmacol..

[77] Shanahan M., Bingman V.P., Shimizu T., Wild M., Güntürkün O. (2013). Large-scale network organization in the avian forebrain: A connectivity matrix and theoretical analysis. Front. Comput. Neurosci..

[78] Stacho M., Herold C., Rook N., Wagner H., Axer M., Amunts K., Güntürkün O. (2020). A cortex-like canonical circuit in the avian forebrain. Science.

[79] Crook R.J. (2021). Behavioral and neurophysiological evidence suggests affective pain experience in octopus. iScience.

[80] Schnell A.K., Browning H., Crump A., Burn C.C., Birch J. (2026). Sentience in cephalopod molluscs: An updated assessment. Biol. Rev. Camb. Philos. Soc..

[81] Weinhard L., di Bartolomei G., Bolasco G., Machado P., Schieber N.L., Neniskyte U., Exiga M., Vadisiute A., Raggioli A., Schertel A. (2018). Microglia remodel synapses by presynaptic trogocytosis and spine head filopodia induction. Nat. Commun..

[82] Matejuk A., Ransohoff R.M. (2020). Crosstalk between astrocytes and microglia: An overview. Front. Immunol..

[83] Verkhratsky A., Ho M.S., Parpura V. (2019). Evolution of neuroglia. Adv. Exp. Med. Biol..

[84] Poitelon Y., Kopec A.M., Belin S. (2020). Myelin fat facts: An overview of lipids and fatty acid metabolism. Cells.

[85] Oliveira J.F., Araque A. (2022). Astrocyte regulation of neural circuit activity and network states. Glia.

[86] Sharkey K.A., Mawe G.M. (2023). The enteric nervous system. Physiol. Rev..

[87] Banks W.A. (2012). Brain meets body: The blood-brain barrier as an endocrine interface. Endocrinology.

[88] Banks W.A. (2019). The blood-brain barrier as an endocrine tissue. Nat. Rev. Endocrinol..

[89] Safron A. (2021). The radically embodied conscious cybernetic Bayesian brain: From free energy to free will and back again. Entropy.

[90] Lillo Vizin R.C., Ito H., Kopruszinski C.M., Ikegami M., Ikegami D., Yue X., Navratilova E., Moutal A., Cowen S.L., Porreca F. (2024). Cortical kappa opioid receptors integrate negative affect and sleep disturbance. Transl. Psychiatry.

[91] Mellor D.J. (2016). Updating animal welfare thinking: Moving beyond the “Five Freedoms” towards “A Life Worth Living”. Animals.

[92] Mellor D.J., Beausoleil N.J., Littlewood K.E., McLean A.N., McGreevy P.D., Jones B., Wilkins C. (2020). The 2020 Five Domains Model: Including human-animal interactions in assessments of animal welfare. Animals.

[93] Rault J.L., Kells N., Johnson C., Dennis R., Sutherland M., Lay D.C. (2015). Nitrous oxide as a humane method for piglet euthanasia: Behavior and electroencephalography (EEG). Physiol. Behav..

[94] Khajehnejad M., Habibollahi F., Loeffler A., Paul A., Razi A., Kagan B.J. (2025). Dynamic network plasticity and sample efficiency in biological neural cultures: A comparative study with deep reinforcement learning. Cyborg Bionic Syst..

[95] Gidon A., Aru J., Larkum M.E. (2025). Does neural computation feel like something?. Front. Neurosci..

[96] Dupoux E., LeCun Y., Malik J. (2026). Why AI systems don’t learn and what to do about it: Lessons on autonomous learning from cognitive science. arXiv.

[97] Butlin P., Long R., Bayne T., Bengio Y., Birch J., Chalmers D., Constant A., Deane G., Elmoznino E., Fleming S.M. (2026). Identifying indicators of consciousness in AI systems. Trends Cogn. Sci..

[98] Gurnee W., Sofroniew N., Pearce A., Piotrowski M., Kauvar I., Chen R., Soligo A., Bogdan P., Ong E., Wang R. (2026). Verbalizable representations form a global workspace in language models. arXiv.

[99] Solms M., Grimbly S.J., Bassett B., Boonstra E., Hodson R., Kuske N., Mahadew K., Rosman B., van Hoof C., Shock J. (2026). Inferring affective consciousness in an artificial agent: A case study. J. Conscious. Stud..

[100] Candia-Rivera D. (2026). Interoceptive machine framework: Toward interoception-inspired regulatory architectures in artificial intelligence. Phys. Life Rev..

[101] Moreno A., Mossio M. (2015). Biological Autonomy: A Philosophical and Theoretical Enquiry.

[102] Schrum J.P., Zhu T.F., Szostak J.W. (2010). The origins of cellular life. Cold Spring Harb. Perspect. Biol..

[103] Poirazi P., Brannon T., Mel B.W. (2003). Pyramidal neuron as two-layer neural network. Neuron.

[104] London M., Häusser M. (2005). Dendritic computation. Annu. Rev. Neurosci..

[105] Borisyuk R., Al Azad A.K., Conte D., Roberts A., Soffe S.R. (2011). Modeling the connectome of a simple spinal cord. Front. Neuroinform..

[106] Le Douarin N.M., Dupin E. (2012). The neural crest in vertebrate evolution. Curr. Opin. Genet. Dev..

[107] Newgreen D.F., Dufour S., Howard M.J., Landman K.A. (2013). Simple rules for a simple nervous system? Molecular and biomathematical approaches to enteric nervous system formation and malformation. Dev. Biol..

[108] Hameroff S. (2022). Consciousness, cognition and the neuronal cytoskeleton-a new paradigm needed in neuroscience. Front. Mol. Neurosci..

[109] Klatzmann U., Doerig A. (2026). What biology can, and cannot, tell us about conscious AI. Trends Open.

[110] Ladas A.I., Gravalas T., Stoneham T., Frantzidis C.A. (2023). Towards a hybrid approach to unveil the Chimaira of neurosciences: Philosophy, aperiodic activity and the neural correlates of consciousness. Front. Hum. Neurosci..

[111] Rouleau N., Levin M. (2026). Brains and where else? Mapping theories of consciousness to unconventional embodiments. Philos. Trans. A Math. Phys. Eng. Sci..

[112] Zaremba B., Fallahshahroudi A., Schneider C., Schmidt J., Sarropoulos I., Leushkin E., Berki B., Van Poucke E., Jensen P., Senovilla-Ganzo R. (2025). Developmental origins and evolution of pallial cell types and structures in birds. Science.

[113] Nawroth C., Langbein J., Coulon M., Gabor V., Oesterwind S., Benz-Schwarzburg J., von Borell E. (2019). Farm animal cognition-linking behavior, welfare and ethics. Front. Vet. Sci..

[114] Donald R.D., Healy S.D., Lawrence A.B., Rutherford K.M. (2011). Emotionality in growing pigs: Is the open field a valid test?. Physiol. Behav..

[115] Reep R.L., Finlay B.L., Darlington R.B. (2007). The limbic system in mammalian brain evolution. Brain Behav. Evol..

[116] Zhao Z.D., Zhang L., Xiang X., Kim D., Li H., Cao P., Shen W.L. (2023). Neurocircuitry of predatory hunting. Neurosci. Bull..

[117] Fardell L.L., Pavey C.R., Dickman C.R. (2020). Fear and stressing in predator-prey ecology: Considering the twin stressors of predators and people on mammals. PeerJ.

[118] Carbone L. (2020). Do prey species hide their pain? Implications for ethical care and use of laboratory animals. J. Appl. Anim. Ethics Res..

[119] Rørvang M.V., Nielsen B.L., McLean A.N. (2020). Sensory abilities of horses and their importance for equitation science. Front. Vet. Sci..

[120] Harvey A.M., Beausoleil N.J., Ramp D., Mellor D.J. (2023). Mental experiences in wild animals: Scientifically validating measurable welfare indicators in free-roaming horses. Animals.

[121] Lencioni G.C., de Sousa R.V., de Souza Sardinha E.J., Corrêa R.R., Zanella A.J. (2021). Pain assessment in horses using automatic facial expression recognition through deep learning-based modelling. PLoS ONE.

[122] Dalla Costa E., Pascuzzo R., Leach M.C., Dai F., Lebelt D., Vantini S., Minero M. (2018). Can grimace scales estimate the pain status in horses and mice? A statistical approach to identify a classifier. PLoS ONE.

[123] Barreto da Rocha P., Driessen B., McDonnell S.M., Hopster K., Zarucco L., Gozalo-Marcilla M., Hopster-Iversen C., Esteves Trindade P.H., Gonzaga da Rocha T.K., Taffarel M.O. (2021). A critical evaluation for validation of composite and unidimensional postoperative pain scales in horses. PLoS ONE.

[124] Foris B., Sheng K., Dürnberger C., Oczak M., Rault J.L. (2025). AI for One Welfare: The role of animal welfare scientists in developing valid and ethical AI-based welfare assessment tools. Front. Vet. Sci..

[125] Yakhlef V., Magalhães-Sant’Ana M., Pereira A.L., Azevedo A. (2025). A global survey on the perception of conservationists regarding animal consciousness. Animals.

[126] Broomé S., Feighelstein M., Zamansky A., Lencioni G.C., Andersen P.H., Pessanha F., Mahmoud M., Kjellström H., Salah A.A. (2023). Going deeper than tracking: A survey of computer-vision based recognition of animal pain and emotions. Int. J. Comput. Vis..

[127] Ferdinandy B., Gerencsér L., Corrieri L., Perez P., Újváry D., Csizmadia G., Miklósi Á. (2020). Challenges of machine learning model validation using correlated behaviour data: Evaluation of cross-validation strategies and accuracy measures. PLoS ONE.

[128] Wilson O., Schoeman D., Bradley A., Clemente C. (2025). Practical guidelines for validation of supervised machine learning models in accelerometer-based animal behaviour classification. J. Anim. Ecol..

[129] Riaz M.U., O’Grady L., McAloon C.G., Logan F., Gormley I.C. (2025). Comparison of machine learning and validation methods for high-dimensional accelerometer data to detect foot lesions in dairy cattle. PLoS ONE.

[130] Tejani A.S., Klontzas M.E., Gatti A.A., Mongan J.T., Moy L., Park S.H., Kahn C.E., CLAIM 2024 Update Panel (2024). Checklist for Artificial Intelligence in Medical Imaging (CLAIM): 2024 Update. Radiol. Artif. Intell..

[131] Ong Ly C., Unnikrishnan B., Tadic T., Patel T., Duhamel J., Kandel S., Moayedi Y., Brudno M., Hope A., Ross H. (2024). Shortcut learning in medical AI hinders generalization: Method for estimating AI model generalization without external data. npj Digit. Med..

[132] Van Calster B., Wynants L., Verbeek J.F.M., Verbakel J.Y., Christodoulou E., Vickers A.J., Roobol M.J., Steyerberg E.W. (2018). Reporting and interpreting decision curve analysis: A guide for investigators. Eur. Urol..

[133] Kompa B., Snoek J., Beam A.L. (2021). Second opinion needed: Communicating uncertainty in medical machine learning. npj Digit Med..

