# Supplementary material for: Artificial Intelligence and Metazoan Nervous Systems: From Local Processing to Valenced States of the Organism and the Inference of Suffering
Source: Animals (Basel). 2026 Oct 26;16(21):3376. doi: 10.3390/ani16213376 (PMC13761609; doi:10.3390/ani16213376)
Supplement: Supplementary file 1 [file animals-16-03376-s001.zip › animals-4595693-supplementary.pdf]

Supplementary Material S1
Review Protocol and Epistemological Appraisal Framework

All references cited in this Supplementary Material are included in the reference list of the main manuscript.

For: Artificial Intelligence and Metazoan Nervous Systems: From Local Processing to Valenced States of the Organism and the Inference of Suffering

Protocol version 3.4, 19 October 2026. Scopus (Elsevier, Amsterdam, The Netherlands) searches and exports completed on 16 October 2026; search history incorporates author-confirmed dates, search fields and export provenance. Targeted PubMed (National Library of Medicine, Bethesda, MD, USA) coverage audit conducted on 22 July 2026.

This Supplementary Material documents literature identification and appraisal for a structured critical narrative review. It includes the targeted PubMed coverage audit and the supplementary Scopus literature check undertaken during revision. It does not represent a preregistered systematic review or meta-analysis. Version 3.4 incorporates the supplied Scopus search history and distinguishes author-reported metadata from independently parsed export counts. The recorded PubMed search strings, date and retrieval counts remain unchanged. Representative publications in Table S1 are identified by author and reference number; their full references appear in the reference list of the main manuscript.

S1. Review purpose and development

The review addresses an inferential problem rather than a single intervention or diagnostic test: which biological observations make it increasingly plausible that local neural processing has become an integrated, valenced and potentially conscious state of an animal, and which observations remain compatible with complex processing without feeling. The work developed in two linked phases. Initial exploratory reading identified candidate mechanisms, conceptual disagreements and recurrent errors of inference. A subsequent structured verification phase used defined concept blocks, sentinel publications and a claim-level appraisal procedure to re-examine the propositions and citations used in the manuscript.

Four propositions guide the appraisal. First, local detection, reflex organisation, arousal, learning and adaptive output are not individually sufficient to establish conscious experience. Second, evidence of modulation, valuation, flexible integration and organismal consequences addresses distinct inference questions; support for one does not automatically establish the others. Third, functional implementation in an artificial system does not independently establish subjective experience, nor does it show that the implemented function is unnecessary in a conscious biological system. Fourth, an aversive stimulus alone does not establish suffering. Welfare assessment considers evidence of negative valuation alongside acute severity, duration, recurrence, controllability, functional consequences and recovery, without requiring persistence or impairment for an acute experience to be seriously adverse.

S2. Scope and information sources

The principal evidence domains were evolutionary neurobiology, cellular and systems neuroscience, affective and comparative neuroscience, consciousness science, animal pain and welfare science, veterinary and behavioural research, network science, biological autonomy and artificial neural-network research.

Mammalian and avian evidence was emphasised for welfare application. Invertebrate, non-neural, artificial and cultured-neuronal systems were included as mechanistic or functional comparison cases. Biological neuronal cultures were distinguished from artificial neural networks. Neither class was presumed to provide proof of the absence of experience.

Exploratory literature identification used PubMed/Medical Literature Analysis and Retrieval System Online (MEDLINE), Scopus, Google Scholar (Google LLC, Mountain View, CA, USA) and publisher resources, together with backward reference checking and forward citation tracking. No fixed lower publication date was imposed, allowing inclusion of foundational pharmacological, neurophysiological and theoretical sources. Source verification continued during revision. Five supplementary Scopus searches were executed on 16 October 2026, and their results were exported and processed on the same date. Section S8a documents the search history, export details and subsequent appraisal.

Comparative cases were selected for their ability to clarify inference rather than for taxonomic representativeness. Amphioxus distinguishes organised locomotor control from untested claims about valuation. Zebrafish studies address pharmacological modulation, responses to ambiguity and internal-state regulation under different conditions; they are not combined as proof of a single persistent negative state. Birds and mammals provide anatomical comparisons relevant to integration. Octopus provides a comparison beyond vertebrate architecture, with behavioural and peripheral neurophysiological findings appraised separately. Differences in evidence across taxa are not treated as a ranking of subjective experience.

S3. Derivation and validation of search terms

Keywords were generated by translating each research proposition and each biological stage of the framework into a searchable concept block. Terms were expanded using synonyms, spelling variants, author keywords and controlled indexing terms encountered in relevant titles and abstracts. A single large search expression was avoided because it would combine disciplines with different vocabularies and generate large quantities of irrelevant material. Instead, focused searches were run for each mechanistic question. In the search expressions, AI stands for artificial intelligence.

Table S1. Conceptual search templates, core concept blocks and representative publications.

Search domain	Core concept block	Generic Boolean search logic	Representative publications
Evolutionary origins	Nervous-system origins, cellular excitability, secretion, bioelectricity and early neural organisation	(“nervous system evolution” OR “elementary nervous systems” OR “neuron evolution” OR “neural origins” OR bioelectric*) AND (metazoa* OR animal* OR multicellular)	Arendt [19,23]; Arendt et al. [18]; Moran et al. [22]
Distributed state modulation	Volume transmission, diffuse neuromodulation, neuropeptides, monoamines and extrasynaptic signalling	(“volume transmission” OR “diffuse neuromodulation” OR extrasynaptic OR neuropeptide* OR monoamine*) AND (brain OR neuron* OR “nervous system”)	Agnati et al. [32]; Gianni and Pasqualetti [33]; Hökfelt et al. [34]
Affect and conscious access	Affect, valence, pain, emotion, sentience, consciousness, recurrence and wider availability	(affect OR valence OR emotion OR feeling OR pain OR suffering OR sentience OR consciousness) AND (recurrent OR integration OR broadcasting OR “global availability” OR access)	Mashour et al. [43]; Paul et al. [52]; Birch et al. [66]
Flexible and contextual processing	Memory, anticipation, learning, contextual use, behavioural flexibility and network topology	(memory OR anticipation OR “flexible behaviour” OR “behavioral flexibility” OR generalisation OR contextual OR “network topology”) AND (animal* OR neural OR consciousness OR affect)	Sergent et al. [44]; How et al. [69]; Papo et al. [68]
Organismal coupling	Autonomic, endocrine, metabolic, immune, interoceptive and recovery consequences	(affect OR pain OR stress OR suffering) AND (autonomic OR endocrine OR metabolic OR immune OR interoception OR allostasis OR recovery OR sleep OR feeding)	Quadt et al. [58]; Banks [87,88]; Sharkey and Mawe [86]
Artificial and biological functional comparisons	Artificial networks and cultured neuronal systems; recurrence, diffusion, neuromodulation and adaptive learning	("artificial neural network*" OR AI OR "spiking neural network*" OR "reinforcement learning" OR "neuronal culture*") AND (recurrence OR diffusion OR neuromodulation OR integration OR adaptive OR modular*)	Velez and Clune [39]; Barretto-Bittar et al. [40]; Gidon et al. [95]; Dupoux et al. [96]
Welfare inference and AI validation	Pain indicators, facial expression, behavioural output, biomarker specificity, classifier validity, external validation, calibration, subgroup performance, distribution or domain shift, model uncertainty, decision thresholds and consequences of classification errors	(animal* OR equine OR livestock) AND (pain OR welfare OR suffering) AND (proxy OR biomarker OR "facial expression" OR classifier OR "machine learning" OR validation OR "external validation" OR calibration OR "distribution shift" OR "domain shift" OR uncertainty OR threshold*)	Sneddon et al. [6]; Lencioni et al. [121]; Foris et al. [124]

Table S1 lists conceptual search templates and representative publications. These templates describe the topics and vocabulary of the exploratory work; they are not a substitute for the exact queries executed in each database. Key publications informed vocabulary development and citation tracking, but complete retrieval of all representative publications by the supplementary Scopus searches has not been demonstrated. Their designation gives them no automatic evidential priority.

S4. Eligibility, provenance and source disposition

Peer-reviewed primary studies, systematic or critical reviews, and clearly identified theoretical analyses were eligible when they contributed directly to one of the research propositions. Narrative reviews and scholarly books were retained for

definitions, historical synthesis or conceptual distinctions but were not substituted for primary evidence when a specific experimental claim was made. Preprints were considered only for rapidly developing artificial-network research, were labelled explicitly and were used as mechanistic or hypothesis-generating evidence rather than as sole support for a central biological conclusion.

A source was not excluded merely because it could not establish feeling or consciousness. Instead, its permissible role was restricted to what it directly supported. An anatomical study could establish structural variation without measuring experience; a facial-expression study could establish a movement-context association without directly measuring pain; and an artificial-network paper could demonstrate functional implementation without addressing phenomenology. Table S2 summarises the source dispositions used.

Table S2. Source disposition categories and their typical use.

Disposition	Meaning	Typical use
Core evidence	Directly supports a central biological or inferential proposition with appropriate design and scope.	Construction of the framework.
Supporting evidence	Strengthens a proposition but is not independently sufficient or fully specific.	Contextual, comparative or convergent support.
Conceptual evidence	Clarifies terminology, theory, assumptions or limits without independently establishing an empirical claim.	Definitions and hypothesis development.
Not used for the proposed inference	Valid observation but unsuitable for the stronger interpretation, or derivative, redundant or insufficiently transparent material.	May be cited only for a narrower point or omitted.

S5. Claim-level extraction and appraisal

Appraisal focused on individual manuscript claims rather than assigning one overall quality score to each publication. Table S3 lists the fields used to organise claim–evidence comparisons and guide targeted source checks. It is an appraisal framework, not a claim that a complete matrix was populated for every publication or every sentence. Construct validity concerns whether an indicator reflects the state it is intended to assess. Complementary validation tests increase confidence when they address different potential sources of error, as discussed by Mason [16].

Table S3. Fields used for claim-level extraction and appraisal.

Field	Information recorded
Source and manuscript location	Bibliographic source, reference number and sentence or section supported.
Direct measurement	The variable actually observed, manipulated or calculated.
Target construct	Detection, arousal, valuation, emotion, feeling, consciousness, suffering or welfare.
Proxy or approximation	Whether the measure is associated with an inaccessible target or estimates its magnitude.
Biological specificity	Other plausible causes of the same observation.
Species and context validation	Relevance to the taxon, state, experimental context and time scale.
Alternative explanations	Whether competing mechanisms were discussed or tested.
Causal and temporal strength	Cross-sectional, longitudinal, intervention, reversal, lesion, stimulation or treatment evidence.
Independence of convergence	Whether converging findings constrain different alternatives, rather than providing correlated measures of the same response.
Artificial or formal functional comparison	Whether the same function occurs without evidence of feeling.
Model generalisability and uncertainty	Whether performance has been assessed across relevant animals or subgroups and outside the development distribution, including external validation, calibration, distribution or domain shift, and the handling of uncertain or out-of-domain inputs.
Decision consequence	Whether threshold choice and false-positive and false-negative classifications are interpreted in relation to the intended welfare use and their potentially unequal biological consequences.
Permissible conclusion	The strongest inference proportionate to the data.

Field	Information recorded
Inferential limit	What the study cannot establish.
Framework role	Capacity, occurrence, organismal burden or confidence.
Disposition and qualitative evidence role	Core, supporting, conceptual or not used for the proposed inference; no numerical or ordinal evidence grade was assigned.

S6. Appraisal questions and decision rules

The questions in Table S4 guided appraisal. They structure scientific judgement rather than constitute a validated scoring algorithm. A negative or unresolved answer may restrict the inference supported by a source without requiring its exclusion. Missing evidence, an uninformative negative finding and evidence contradicting a claim were distinguished.

Table S4. Appraisal questions and decision rules.

Step	Appraisal question	Decision consequence
1	What was directly measured or manipulated?	Record the observation without an emotion or consciousness label.
2	What internal construct is inferred?	Separate detection, arousal, valuation, feeling, consciousness and suffering.
3	Is the construct defined consistently?	If not, restrict interpretation to the measured variable.
4	Is the measure a proxy or a genuine approximation?	Do not convert association into a numerical estimate of subjective magnitude.
5	Is the measure biologically specific?	Downgrade claims when exertion, novelty, illness, restraint, reward or fear offer alternatives.
6	Was the measure validated in the relevant species and context?	Treat unvalidated transfer as hypothesis-generating.
7	Were competing explanations tested?	Increase claim-specific confidence when treatment, reversal, lesion, stimulation or a controlled contrast provides causal leverage.
8	Do complementary evidence streams address different alternative explanations?	Give greater weight to convergence that limits different sources of error; correlated indicators alone do not supply independent validation.
9	Can an artificial or formal system implement the same function without providing independent evidence of subjective experience?	Functional implementation alone does not establish feeling or exclude a contribution of the corresponding mechanism in a biological system.
10	Does the conclusion exceed the design?	Narrow claims that move from structure or performance directly to experience.
11	Which inferential question is answered?	Assign the evidence to capacity, occurrence, burden or confidence.
12	What is the final role in the synthesis?	Retain as core, supporting or conceptual evidence, or do not use for the proposed inference.
13	Are welfare concern and confidence in the inference reported separately?	State welfare concern and evidential confidence independently; do not combine them into a single score.

S7. Synthesis and safeguards against interpretive bias

The synthesis is organised around local detection, distributed modulation, affective valuation, recurrent and flexible integration, and acute or sustained organismal consequences. These domains are not treated as successive levels of consciousness. Species-level capacity is distinguished from evidence of occurrence in an individual and from the welfare significance of the inferred state. Conflicting findings and plausible alternatives are considered without treating one theory of consciousness as decisive.

The synthesis considered plausible alternatives and conflicts in evidence. Human-like expression alone was not treated as stronger evidence, nor was its absence treated as evidence against experience. Source provenance and declared conflicts were considered where relevant. Duplicate records were merged when matching identifiers supported this decision; possible

duplicates without sufficient identifier agreement were retained. AI-generated appraisals were treated as draft recommendations requiring author judgement.

For studies involving machine-learning classification or AI-assisted welfare decision support, technical performance was considered separately from biological construct validity. Overall accuracy was not treated as sufficient where performance differed among animals, breeds, clinical conditions, management settings or recording environments. Where relevant, external validation, calibration, subgroup performance, distribution shift and model uncertainty were considered. Decision thresholds were interpreted in relation to intended use and to the potentially unequal biological consequences of false-positive and false-negative classifications. Agreement with expert labels was treated as evidence of label reproduction rather than independent validation of pain, affect or suffering.

AI assistance and author responsibility

Generative AI tools supported keyword development, record parsing, deduplication, topic prioritisation, bibliography comparison and preparation of draft appraisal rationales. They also helped organise claim–evidence tables, compare abstracts and selected source passages with manuscript claims, and edit language. An author ran the PubMed searches; Scopus exports were supplied by Çağrı Çağlar Sinmez. AI-assisted recommendations informed the author's review of proposed additions. Source interpretation, inclusion and final manuscript wording remain author decisions. This record does not claim independent duplicate human screening or complete full-text checking of all retrieved records.

S8. Targeted PubMed coverage audit

A targeted coverage audit was conducted in PubMed/MEDLINE on 22 July 2026 to describe how often four prespecified domains co-occurred within PubMed indexing and abstracts: domestic-animal welfare, welfare-relevant affect or suffering, neurobiological evidence and inferential validation. The audit was performed after the initial conceptual synthesis and was retained as a narrow coverage observation rather than as the primary method of source discovery. It was not designed to establish the absence of related frameworks, estimate their prevalence or support the manuscript's novelty claim. No restrictions were applied for publication date, language, article type or full-text availability.

Four concept blocks were entered separately in PubMed Advanced Search and combined through the search history. Medical Subject Headings (MeSH) terms were supplemented with title-and-abstract terms and proximity operators. The exact blocks used in the valid final search history are reproduced below, and the retrieval counts of their combinations are given in Table S5. In the search strings, [Mesh] denotes a MeSH term, [tiab] the title/abstract field, EEG electroencephalography, fMRI functional magnetic resonance imaging and ~n a proximity operator.

Block A - domestic and farm animals

```
("Animals, Domestic"[Mesh] OR "Livestock"[Mesh] OR "Horses"[Mesh] OR "Cattle"[Mesh] OR "Swine"[Mesh] OR "Sheep"[Mesh] OR "Goats"[Mesh] OR "farm animal"[tiab] OR "farm animals"[tiab] OR livestock[tiab] OR "domestic animal"[tiab] OR "domestic animals"[tiab] OR horse[tiab] OR horses[tiab] OR equine[tiab] OR donkey[tiab] OR donkeys[tiab] OR cattle[tiab] OR bovine[tiab] OR cow[tiab] OR cows[tiab] OR calf[tiab] OR calves[tiab] OR pig[tiab] OR pigs[tiab] OR porcine[tiab] OR swine[tiab] OR sheep[tiab] OR ovine[tiab] OR goat[tiab] OR goats[tiab] OR caprine[tiab])
```

Block B - welfare, affect and suffering

```
("Animal Welfare"[Mesh] OR "animal welfare"[tiab] OR suffering[tiab] OR distress[tiab] OR sentien*[tiab] OR conscious*[tiab] OR "affective state"[tiab] OR "affective states"[tiab] OR "negative affect"[tiab] OR "positive affect"[tiab] OR emotion*[tiab] OR pain[tiab] OR fear[tiab] OR frustration[tiab] OR aversion[tiab] OR aversive[tiab])
```

Block C - neurobiological evidence

```
("Nervous System Physiological Phenomena"[Mesh] OR "Electrophysiology"[Mesh] OR "Electroencephalography"[Mesh] OR "Brain Mapping"[Mesh] OR neurophysiolog*[tiab] OR neurobiolog*[tiab] OR neuroanatom*[tiab] OR neuroimaging[tiab] OR electrophysiolog*[tiab] OR electroencephalograph*[tiab] OR EEG[tiab] OR fMRI[tiab] OR "brain activity"[tiab:~0] OR "brain activation"[tiab:~0] OR "functional connectivity"[tiab:~0] OR "neural correlate"[tiab:~1] OR "neural correlates"[tiab:~1])
```

Block D - inferential framework and validation

```
("animal welfare framework"[tiab:~2] OR "welfare assessment framework"[tiab:~2] OR "welfare inference"[tiab:~2] OR "suffering assessment"[tiab:~2] OR "suffering inference"[tiab:~2] OR "affective state assessment"[tiab:~2] OR "affective state inference"[tiab:~2] OR "evidence integration"[tiab:~0] OR "weight of evidence"[tiab:~0] OR "construct validity"[tiab:~0] OR "criterion validity"[tiab:~0] OR "convergent validity"[tiab:~0] OR "decision framework"[tiab:~1] OR "evidential confidence"[tiab:~1] OR ((framework[tiab] OR model[tiab]) AND (inferen*[tiab] OR validat*[tiab]) AND (welfare[tiab] OR suffering[tiab] OR affective[tiab])))
```

Table S5. PubMed search intersections and retrieval counts.

Combination	Interpretive purpose	Records
A AND B	Domestic animals plus welfare or affect	31,514

Combination	Interpretive purpose	Records
A AND B AND C	Addition of neurobiological evidence terms	3,607
A AND B AND D	Addition of framework, inference or validation terms	249
A AND B AND C AND D	Full four-domain intersection	25
B AND C AND D	Four-domain logic without the domestic-animal block	2,416

All 25 records in the full four-domain intersection were exported and screened by one author at title-and-abstract level. No duplicate screening or formal inter-rater agreement was undertaken. For descriptive classification within this narrow intersection, a record was considered to combine all four prespecified elements only if it substantively addressed (i) a domestic or farm-animal population, (ii) welfare-relevant affect, pain, suffering or consciousness, (iii) neurobiological or neurophysiological evidence, and (iv) an explicit method for turning that evidence into an inference or decision. This operational classification was not used to draw conclusions about the wider literature. Table S6 summarises the screening outcome.

Table S6. Screening outcome of the records retrieved by the four-block PubMed search.

Screening category	Operational description	Records
Combined all four substantive elements	Substantively addressed the domestic-animal, welfare-relevant, neurobiological and explicit inference or decision elements	0
Related welfare-framework application	Explicitly linked measurable physical or functional indicators to cautiously inferred mental experience	1
Restricted bridge from proxy to affect or consciousness	Addressed one bounded inferential bridge, such as place aversion, EEG-defined loss of consciousness or emotionality	3
Clinical, psychometric or intervention study	Pain-scale validation, grimace-scale analysis, analgesic evaluation or other condition-specific measurement	20
Terminological false positive	Search terms were present but the paper did not address animal-welfare inference	1

Within this narrowly defined four-domain intersection, 20 records were clinical, psychometric or intervention studies; three addressed a restricted bridge from a proxy to affect or consciousness; one applied the Five Domains Model; and one was a terminological false positive. None met the operational substantive criteria for all four elements. This result is reported only as a coverage observation about the prespecified PubMed intersection. It does not establish that related or partially integrative frameworks are absent, estimate the prevalence of such frameworks or support the manuscript's novelty claim. Relevant component literature and related conceptual frameworks occur outside this intersection and were incorporated through the broader narrative search and citation chaining.

S8a. Supplementary Scopus literature check during revision

Purpose and processing

The supplementary check sought consequential omissions in biological, comparative, technical and inferential literature and assessed whether additional sources supported, qualified or challenged the manuscript. It did not reopen every claim or attempt an exhaustive systematic review.

Five exports contained 15,512 record appearances. Deduplication by Scopus Electronic Identifier (EID) produced 15,374 records; merging 14 additional pairs with matching digital object identifiers (DOIs) produced a working set of 15,360 records. Records lacking DOIs were retained using their Scopus identifiers. Possible title matches without sufficient identifier agreement were not automatically merged.

Automated topic prioritisation and comparison with the manuscript bibliography guided targeted appraisal. Forty selected records received individual appraisal, mainly from abstracts; one was assessed from title information only and was not used to substantiate a manuscript claim. Five publications were proposed for additions. Relevant full-text passages were checked for Schnell et al. [80], Mason [16], Ede et al. [62] and Riaz et al. [129]; the proposed use of Brown and Birch [56] was limited

to what their abstract supported. These contributions were discussed with the lead author during revision. This does not imply full-text appraisal of the entire collection.

Export inventory

S01: New S01.txt - 1,274 record appearances.

S02: New S02.txt - 5,378 record appearances.

S03: New S03.txt - 6,187 record appearances.

S04: New S04.txt - 1,945 record appearances.

S05: New S05 (1).txt - 728 record appearances.

The parsed file counts agree with the Scopus totals reported by the search author in A.Search History SCOPUS D01.docx, supplied on 17 October 2026. The author reports that all results were exported for each search and that no filters were applied on the results page before export. These are author-reported search-interface details; the result screens were not independently inspected. The files were received and processed on 16 October 2026. The retained processing record includes the deduplicated record log, duplicate aliases, selected appraisals, source-file checksums and processing script.

Contributions to the revised manuscript

Schnell et al. [80], DOI 10.1002/brv.70125 - Section 5.5: qualifies the octopus relief-preference inference by identifying the missing matched acid-plus-saline conditioning control; distinguishes evidence strength across cephalopod groups from the intensity of experience.

Brown and Birch [56], DOI 10.1098/rstb.2024.0309 - Section 5.3: explains why the relevance of motivational trade-offs depends on their nature and neural mechanisms. The abstract supports this bounded conceptual point; full-text verification was not completed.

Mason [16], DOI 10.1177/00236772251380871 - Section 2.4: supports complementary construct-validation tests with differing assumptions and potential sources of error.

Ede et al. [62], DOI 10.3168/jdsc.2022-0241 - Section 5.3: adds a negative place-conditioning finding after castration with multimodal pain control. Absence of avoidance does not establish absence of pain. This is distinct from the disbudding comparison [61].

Riaz et al. [129], DOI 10.1371/journal.pone.0325927 - Section 10: illustrates validation on farms excluded from model training and the distinction between detecting veterinary-diagnosed lesions and measuring experienced pain. Greater performance variability and calibration differences were reported; a uniform significant reduction in the area under the receiver operating characteristic curve (AUC) was not established.

Confirmed Scopus search history

The search log and subsequent author confirmations document the search and export date as 16 October 2026 and the search field as TITLE-ABS-KEY for all five searches. The expressions below retain the supplied terms and Boolean combinations. Duplicated quotation marks and extraneous outer punctuation introduced during transcription have been normalised, and the author-confirmed field code has been restored where it was omitted from the supplied copy. These editorial corrections do not represent new searches.

Common export settings

Database: Scopus. All five searches used TITLE-ABS-KEY. The search author reports that no filters were applied on the results pages before export, that each complete displayed result set was exported and that no result ranges were omitted.

Export fields selected: Authors, Author full names, Author(s) ID, Title, Year, Source title, Volume, Issue, Art. No., Page start, Page end, Cited by, DOI, Link, Abstract, Document Type, Publication Stage, Open Access, Source, EID. (Export in comma-separated values (CSV) format, saved as .txt. Affiliations, keywords and references were not selected.)

Search and export date for S01–S05: 16 October 2026.

S01 search record
Total results displayed by Scopus: 1,274

Export filename(s): New S01.txt

Exported range: All results (1-1,274)

Was the complete result set exported? Yes. The file contains 1,274 records with 1,274 unique EIDs, which matches the Scopus total of 1,274.

Search expression:

TITLE-ABS-KEY((animal* OR vertebrate* OR invertebrate* OR bird* OR avian OR fish OR cephalopod*) AND (sentien* OR conscious* OR "affective state*" OR "emotional state*" OR valence OR "animal emotion*") AND (framework* OR "convergent evidence" OR "evidence integration" OR "construct validity" OR "comparative neuroscience"))

S01 was run three times with the identical query, corresponding to history entries 31, 32 and 33; each returned 1,274 results. The third run supplied the export imported for analysis. Only this single S01 export was included in the processing counts.

S02 search record
Total results displayed by Scopus: 5,378

Export filename(s): New S02.txt

Exported range: All results (1-5,378)

Was the complete result set exported? Yes. The file contains 5,378 records with 5,378 unique EIDs, which matches the Scopus total of 5,378.

Search expression:

TITLE-ABS-KEY(("volume transmission" OR neuromodulat* OR neuropeptide* OR serotonerg* OR dopaminerg*) AND ("internal state*" OR "persistent state*" OR "state-dependent" OR "state dependent" OR arousal OR valence) AND (animal* OR zebrafish OR mouse OR mice OR lamprey OR octopus OR drosophila OR brain OR neural))

S03 search record
Total results displayed by Scopus: 6,187

Export filename(s): New S03.txt

Exported range: All results (1–6,187)

Was the complete result set exported? Yes. The file contains 6,187 records with 6,187 unique EIDs, which matches the Scopus total of 6,187.

Search expression:

TITLE-ABS-KEY((animal* OR livestock OR horse* OR equine OR cattle OR calf OR calves OR pig* OR sheep OR goat* OR zebrafish OR octopus OR mouse OR mice) AND ("conditioned place preference" OR "conditioned place aversion" OR "judgement bias" OR "judgment bias"OR "motivational trade-off*" OR "motivational tradeoff*" OR "pain relief") AND (pain OR avers* OR valence OR emotion* OR "negative affect" OR persistence OR recovery OR sleep))

S04 search record
Total results displayed by Scopus: 1,945

Export filename(s): New S04.txt

Exported range: All results (1-1,945)

Was the complete result set exported? Yes. The file contains 1,945 records with 1,945 unique EIDs, which matches the Scopus total of 1,945.

Search expression:

TITLE-ABS-KEY(("artificial intelligence" OR "artificial neural network*" OR "language model*" OR "artificial agent*" OR "spiking neural network*") AND (conscious* OR sentien* OR "subjective experience" OR "affective state*") AND (indicator* OR framework* OR recurrent OR recurrence OR "global workspace" OR neuromodulat* OR homeostas* OR interocept*))

S05 search record

Total results displayed by Scopus: 728

Export filename(s): New S05 (1).txt

Exported range: All results (1-728)

Was the complete result set exported? Yes. The file contains 728 records with 728 unique EIDs, which matches the Scopus total of 728.

Search expression:

TITLE-ABS-KEY((animal* OR livestock OR horse* OR equine OR cattle OR pig* OR sheep OR goat* OR canine OR feline) AND (pain OR welfare OR emotion* OR "affective state*") AND ("machine learning" OR "deep learning" OR "computer vision" OR "automated assessment" OR "automatic recognition") AND (validat* OR "ground truth" OR generalis* OR generaliz* OR "data leakage" OR "domain shift" OR "reference label*"))

S9. Limits of the review procedure

The review remains a structured critical narrative synthesis. The targeted PubMed audit concerns one database and one terminology intersection; it cannot establish absence, prevalence or novelty of related frameworks. Its 25 records were screened by one author without duplicate screening or formal agreement testing. The supplementary Scopus exports underwent automated organisation and prioritisation followed by targeted appraisal of selected records. The full deduplicated collection was not exhaustively screened or appraised record by record, and full texts were checked selectively. Prioritisation may miss relevant sources, including papers using different terminology. The broader searches and citation tracking therefore cannot guarantee that no relevant paper was missed. No meta-analysis, numerical evidence weighting or composite consciousness score was attempted. The framework organises and limits inference; its proposed biological links have not been validated as a complete causal sequence or quantitative scale of experience.
